# Spatial‐Scale Evolutionary Bias Sheds Light on the Latitudinal Diversity Gradient

**DOI:** 10.1002/ece3.74111

**Published:** 2026-07-29

**Authors:** Haoyuan Wu, Donghui Xu, Yonghua Wu

**Affiliations:** ^1^ School of Life Sciences Northeast Normal University Changchun China; ^2^ Jilin Provincial Key Laboratory of Animal Resource and Ecological Security Northeast Normal University Changchun China

**Keywords:** evolutionary bias, experimental evolution, genome sequencing, heterogeneous environments, latitudinal diversity gradient, latitudinal fitness gradient

## Abstract

The latitudinal diversity gradient (LDG) is observed across various biological groups, but its causes remain debated. Here, using *Escherichia coli* as a model system, we experimentally tested whether spatial‐scale evolutionary bias (SSEB)—the tendency for species to evolve adaptively toward areas with higher fitness gains—exists and whether it may contribute to the LDG. We conducted competition and experimental evolution assays between high‐fitness‐gain and low‐fitness‐gain populations. Our results show three key findings: (i) high‐fitness‐gain populations exhibit significantly faster spatial expansion than low‐fitness‐gain populations; (ii) under gene flow, high‐fitness‐gain populations tend to competitively exclude low‐fitness‐gain populations, whereas the reverse effect is minimal; and (iii) experimental populations are more likely to evolve and spread toward regions with higher fitness gains than toward regions with lower gains. These empirical findings collectively demonstrate the occurrence of SSEB. Given that climatic conditions become increasingly favorable from the poles to the equator, a latitudinal fitness gradient is expected for many taxa. Under such a gradient, SSEB would drive biased adaptation toward lower latitudes, either by suppressing adaptation of low‐latitude species to higher latitudes or by promoting evolutionary migration from high to low latitudes. Thus, SSEB may provide a novel evolutionary mechanism contributing to the LDG.

## Graphical abstract

Using *Escherichia coli* as a model species, our experimental findings reveal that populations in high‐fitness‐gain geographic regions undergo rapid expansion, competitively excluding populations from low‐fitness‐gain regions. This results in adaptive evolution that is biased toward areas with higher fitness potential. We propose that this spatial‐scale evolutionary bias plays a crucial role in shaping the LDG.

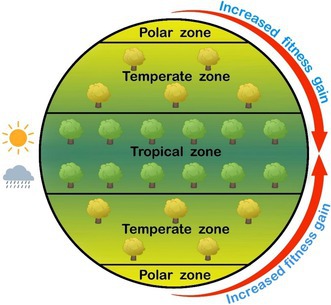

## 1 Introduction

Species diversity on Earth is distributed unevenly. One striking pattern is the latitudinal diversity gradient (LDG), which refers to the increase in species diversity from the poles to the equator. This pattern is observed in nearly all biological taxa (e.g., plants, birds, mammals, amphibians, and fish), with some exceptions (Willig et al. 2003; Hillebrand 2004; Brown 2014). Given that species diversity is influenced by various factors, including ecological, evolutionary, and historical ones (Willig et al. 2003; Mittelbach et al. 2007; Schemske et al. 2009; Raz et al. 2024), the similar spatial distribution patterns of species diversity (Hillebrand 2004) suggest that there may be a single general mechanism driving the LDG (Pianka 1966; Rohde 1992).

Many hypotheses have been proposed to explain the LDG, including ecological and evolutionary perspectives (Pianka 1966; Rohde 1992; Willig et al. 2003; Hillebrand 2004; Mittelbach et al. 2007; Brown 2014). Some climate‐related factors, such as sunlight, temperature, rainfall, and seasonality, which correlate with latitude, have long been considered related to the LDG, but the underlying mechanism remains controversial (Ricklefs 1973; Currie and Paquin 1987; Currie 1991; Willig et al. 2003; Field et al. 2009). The productivity hypothesis suggests that the tropical climate is more productive and can support more individuals, leading to greater species diversity (Connell and Orias 1964; Pianka 1966), or helping to reduce extinction risk (Wright 1983; Turner et al. 1987; Clarke and Gaston 2006; Field et al. 2009). The evolutionary speed hypothesis proposes that higher temperatures in the tropics can accelerate speciation (Rohde 1992). These hypotheses imply that tropical taxa experience relatively higher diversification rates (Mittelbach et al. 2007). However, comparative studies between tropical and extratropical regions show inconsistent results, with relatively higher diversification rates reported in many tropical taxa (Ricklefs 2006; Condamine et al. 2012; Pyron and Wiens 2013; Pyron 2014; Rolland et al. 2014; Fine 2015; Fenton et al. 2023), but other tropical taxa do not show such higher rates (Wiens et al. 2009; Fine 2015; Economo et al. 2018; Egan et al. 2022; Tietje et al. 2022; Willink et al. 2024). These inconsistencies make these hypotheses elusive as general explanations for the LDG (Mittelbach et al. 2007), and additional factors may be involved in the formation of the LDG.

**FIGURE 1:**
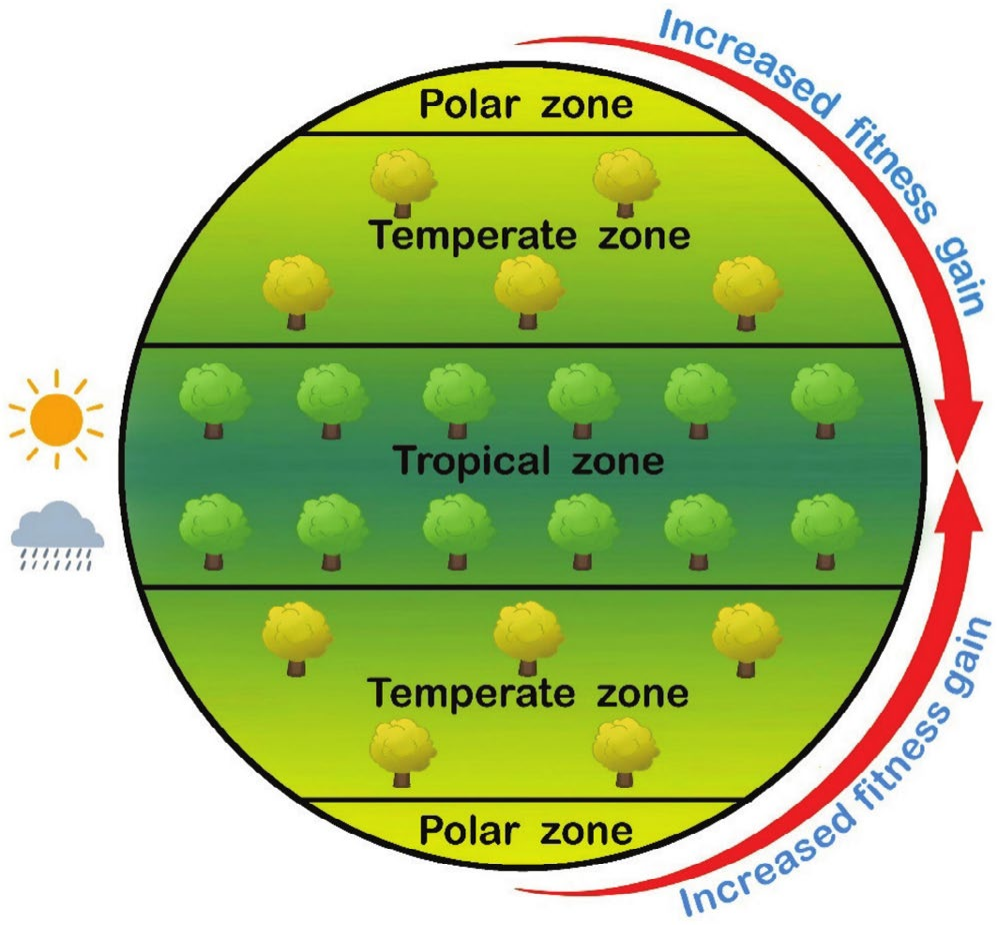
Latitude gradient of fitness gain. Compared to high‐latitude regions, low‐latitude regions (e.g., tropical regions) are characterized by more sunshine, higher temperatures, abundant rainfall, and longer growing seasons. These conditions enable tropical organisms to grow and reproduce quickly, leading to high reproductive output (Hutchinson 1959; Connell and Orias 1964; Wright 1983; Turner et al. 1987; Primack 2010; Brown 2014), and potentially result in an increased fitness gain from polar zones to tropical zones for many groups.

As mentioned above, the Earth's environment is heterogeneous, and climate‐related factors vary continuously with latitude (Dowle et al. 2013; Worm and Tittensor 2018). Compared to high‐latitude regions, which are cold, dry, and have short growing seasons, low‐latitude regions (e.g., the tropics) experience high temperatures, abundant sunlight and precipitation, and long growing seasons. These conditions enhance productivity, allowing tropical organisms to grow and reproduce faster, with higher reproductive output (Hutchinson 1959; Connell and Orias 1964; Wright 1983; Turner et al. 1987; Primack 2010; Brown 2014). Consequently, for many taxa, organisms in low‐latitude regions may experience higher fitness gains than their high‐latitude counterparts, creating a potential latitudinal gradient of increasing fitness gains from the poles to the equator (Figure 1). This latitudinal fitness gradient may exert a potential influence on the formation of the LDG; however, no relevant research on this relationship has been reported to date.

The evolution of species in heterogeneous environments has long been a topic of interest. One of our recent studies revealed a nonspatial evolutionary bias in heterogeneous environments, which refers to the tendency of species to evolve toward the direction with the highest relative fitness gain when multiple evolutionary directions are available (Wu and Wu 2024). Theoretically, evolutionary bias can occur at a spatial scale as well, meaning that species tend to evolve toward areas with high fitness gains—hereafter referred to as spatial‐scale evolutionary bias (SSEB). Regarding SSEB, earlier theoretical studies have shown that in spatially heterogeneous environments, natural selection favors adaptations to more productive habitats over those in poorer habitats (Kawecki 1995; Kawecki et al. 1997). This leads to a contraction in the species' range and biased adaptive evolution toward more favorable source habitats (Holt and Gaines 1992; Holt 2003). Moreover, population geneticists have long recognized that asymmetric gene flow from large, central populations to small, peripheral populations tends to hinder local adaptation and range expansion in the latter, due to genetic swamping and/or disruption of local adaptation (Haldane 1956; E. Mayr 1963; Kirkpatrick and Barton 1997; Lenormand 2002; Bridle and Vines 2007), as supported by empirical studies, including those on walking‐sticks (Bolnick and Nosil 2007), great tits (Postma and van Noordwijk 2005), and threespine stickleback (Hendry et al. 2002). These findings suggest that in spatially heterogeneous environments, the effect of asymmetric gene flow may cause high‐fitness‐gain geographic populations (HFGP) to suppress low‐fitness‐gain geographic populations (LFGP), driving species to evolve in a biased manner toward areas with higher fitness gains, thereby resulting in SSEB.

The potential SSEB provides important insights into the formation of the LDG. Given the latitudinal fitness gradient described above (increasing fitness gains toward the equator), SSEB predicts that tropical (low‐latitude) populations with higher fitness gains will suppress high‐latitude populations with lower fitness gains. This suppression can inhibit adaptive evolution in high‐latitude populations, making it difficult for species to adapt to higher latitudes and expand into those areas. Moreover, high‐latitude populations may even lose their existing adaptability, causing their geographic ranges to contract toward lower latitudes, consistent with theoretical predictions (Haldane 1956; E. Mayr 1963; Kirkpatrick and Barton 1997; Lenormand 2002; Bridle and Vines 2007). Thus, species are ultimately driven to evolve adaptively toward lower latitudes. This evolutionary bias toward lower latitudes could play a critical role in the formation of the LDG, although direct evidence has been lacking.

Given the potential role of SSEB in LDG formation, in this study we used *Escherichia coli* K‐12 GM4792 as a model organism to experimentally test whether SSEB actually occurs. This strain is characterized by its inability to utilize lactose (lac−) due to a 212 bp deletion and a frameshift mutation caused by a single base insertion in the lactose operon, which prevents the lactase gene from being properly expressed (Zhang et al. 2015). However, under appropriate conditions, it can undergo a reversion mutation, evolving into a lactose‐utilizing mutant (lac+). The lac+ and lac− phenotypes can be distinguished on solid media containing X‐gal (5‐bromo‐4‐chloro‐3‐indolyl‐β‐d‐galactopyranoside) and IPTG, where lac+ colonies appear blue and lac− colonies appear white. This blue‐white screening method can be used to differentiate between the two mutations (Wu and Wu 2024). Our recently published study shows that when the ancestral lac− strain, lac‐(ancestor), is placed in M9 medium containing a mixture of abundant sodium acetate and lactose as carbon sources (hereafter referred to as L‐medium), all five lac− experimental populations (L1–5) independently evolved into lac+ after approximately 20 days of experimental evolution (Wu and Wu 2024). The lac+ strain can utilize lactose and gains a higher fitness advantage compared to the lac− strain (Wu and Wu 2024). These two mutant strains, lac+ and lac−, which can be distinguished by color, can be used to produce HFGP and LFGP, thus providing an appropriate experimental system to test SSEB.

Assuming that SSEB indeed exists, it yields three testable predictions: (1) HFGP exhibits relatively rapid population growth and spatial expansion; (2) HFGP tends to exclude LFGP; (3) species are more likely to undergo adaptive evolution toward areas with higher fitness gains, and less likely to undergo adaptive evolution toward areas with lower fitness gains. To test these predictions, we conducted competition experiments between HFGP and LFGP as well as experimental evolution assays. Our laboratory experiments provide strong evidence supporting SSEB, which provides important insight into understanding the formation of the LDG.

## 2 Results

### 2.1 High‐Fitness‐Gain Populations Have Fast Population Increase and Spatial Expansion

**FIGURE 2:**
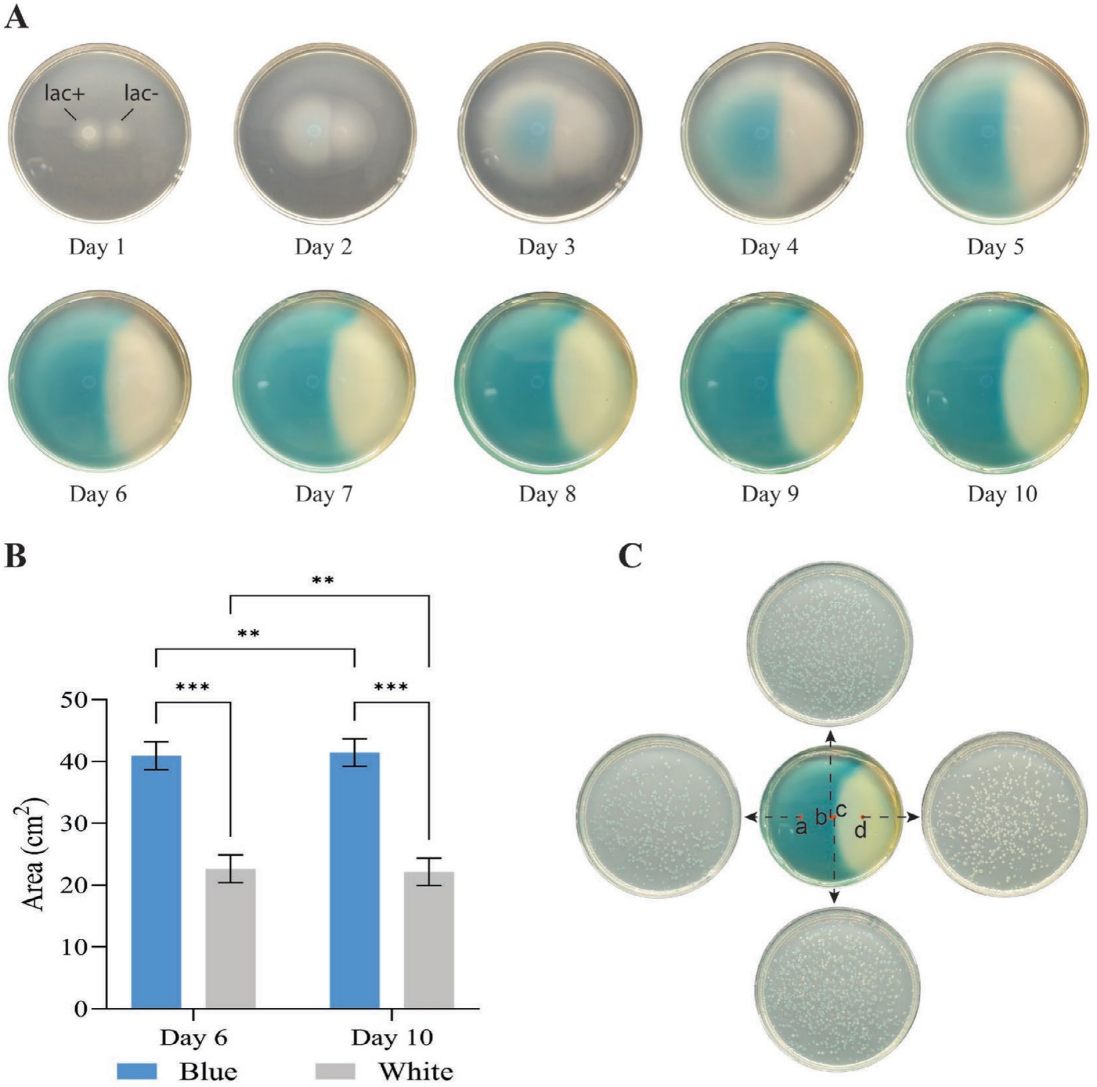
Spatial expansion of lac+ (blue) and lac− (white) on semi‐solid L‐medium plates. (A) Representative plates showing population expansion of lac+ and lac− over time. (B) Distribution areas of lac+ and lac− on Days 6 and 10 from 10 competitive pairs (20 plates total). **p < 0.01, ***p < 0.001 (independent‐samples *t*‐test for between‐type comparisons; paired t‐test for between‐day comparisons). (C) Sampling points for blue‐white screening validation on day 10, and the corresponding plating results are shown alongside the points.

We first tested whether HFGP exhibit faster population growth and spatial expansion than LFGP. To this end, lac+ (blue) and lac− (white) strains were inoculated symmetrically onto semi‐solid L‐medium plates (Figure 2A). In L‐medium, lac+ has a much higher fitness gain than lac− due to its ability to utilize lactose (Wu and Wu 2024), and the two strains can be visually distinguished by blue‐white screening. Equal amounts of the two strains were inoculated on opposite sides of the center of each plate. Our results show that over time, the distribution areas of both lac+ and lac− have increased based on color observation, with the lac+ population expanding and growing at a significantly faster rate than the lac− population (Figure 2A). We measured the distribution areas of both populations on Days 6 and 10. The results (Figure 2B; Table 1) showed that the distribution area of lac+ was significantly larger (approximately 1.8 times) than that of lac− on both Day 6 (*t* = 22.65, *p* < 0.001) and Day 10 (*t* = 24.80, *p* < 0.001). Moreover, from Day 6 to Day 10, the distribution area of lac+ increased by an average of 0.5 cm^2^ (*t* = −4.47, *p* < 0.01), while the distribution area of lac− decreased by 0.5 cm^2^ (*t* = 4.46, *p* < 0.01). These results suggest that the dispersal between high‐fitness‐gain lac+ populations and low‐fitness‐gain lac− populations is asymmetric. The population growth and expansion rate of lac+ are significantly faster than those of lac−, and lac+ has invaded the lac− distribution area, resulting in the competitive exclusion of lac−.

**TABLE 1:** Statistical analyses of blue and white area sizes (cm^2^) in the population expansion experiment. The means (±SD) based on 10 replicate assays are presented.

	Blue	White	*t*	*p*
Day 6	40.94 ± 1.81	22.64 ± 1.81	22.65	< 0.001
Day 10	41.44 ± 1.74	22.15 ± 1.74	24.80	< 0.001
*t*	−4.47	4.46		
*p*	0.0016	0.0016		

The above results are based on color observation of the target strains, lac+ and lac−, and we will next use plating to verify their reliability. We selected five plates for analysis, each representing a sample from one of the five populations (L1–5). Each plate had 4 sampling points, as shown in Figure 2C. After counting a total of 12,668 colonies, the blue‐white screening results (Table S1) showed that in the core area of blue colonies (sampling point a), only blue colonies were found, with no white colonies. In the area near the edge of the blue colonies (sampling point b), the proportion of blue colonies reached 98%. At the boundary line of the blue colonies (sampling point c), the proportion of blue colonies was 83%. In the core area of white colonies (sampling point d), only white colonies were found, with no blue colonies. These results indicate that the blue region is predominantly lac+, while the white region is predominantly lac−. Thus, the blue and white areas can serve as indicators of the distribution areas of lac+ and lac−, respectively, demonstrating the reliability of the above results.

### 2.2 High‐Fitness‐Gain Populations Competitively Exclude Low‐Fitness‐Gain Populations

**FIGURE 3:**
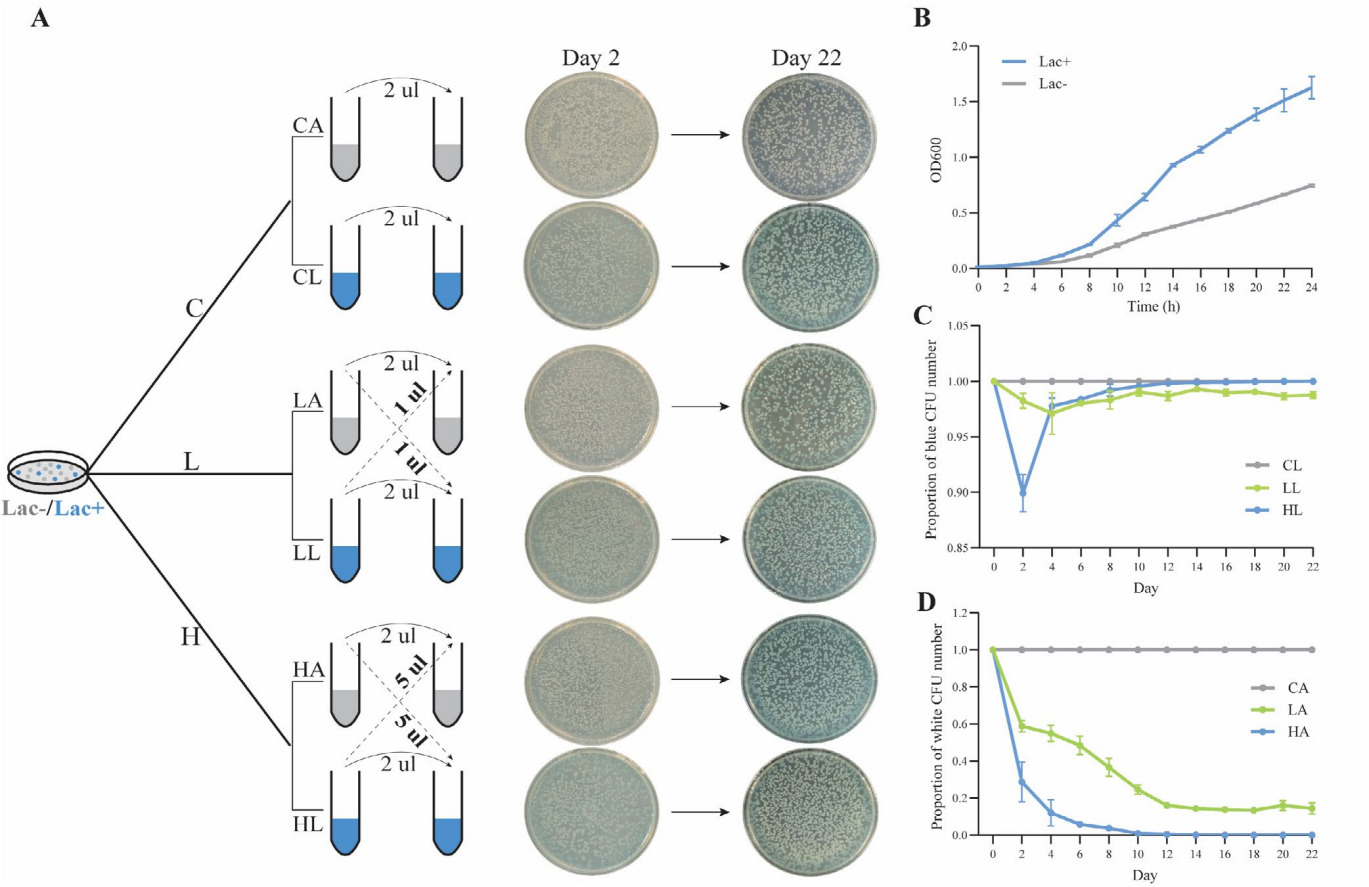
Population dynamics under bidirectional gene flow. (A) Three parallel groups with different levels of gene flow between lac+ (blue) and lac− (gray) populations. Each population had five biological replicates, giving a total of 30 populations across the three groups. Control (C): No gene flow; low gene flow (L): 1 μL culture exchanged per transfer; high gene flow (H): 5 μL culture exchanged per transfer. Representative plating results from Day 2 to Day 22 are shown. (B) Growth curves of lac+ in L‐medium and lac− in A‐medium. (C) Proportion of blue colonies in lac+ populations over time in the three groups. (D) Proportion of white colonies in lac− populations over time in the three groups. For each time point, the mean and standard deviation based on the five replicate populations are shown separately.

We next tested whether HFGP are more likely to outcompete LFGP. To construct these two population types, we isolated lac+ and lac− clones and cultured them in L‐medium and A‐medium, respectively (Figure 3A), with the only difference between the two media being their carbon source composition: L‐medium contains both sodium acetate and lactose as carbon sources, while A‐medium contains only sodium acetate. To verify that lac+ populations have a high fitness gain and lac− populations have a low fitness gain in their respective media, we measured the growth curves of lac+ and lac− in L‐medium and A‐medium, respectively. The results showed that lac+ in L‐medium exhibited almost a two‐fold statistically significant growth advantage over lac− in A‐medium (Figure 3B; Table S2), confirming that lac+ in L‐medium represents HFGP and lac− in A‐medium represents LFGP, which is unsurprising given that lac+ can utilize the high‐energy lactose present in L‐medium, whereas lac− in A‐medium can only metabolize the low‐energy sodium acetate (Wu and Wu 2024). We then exposed the lac+ (HFGP) and lac− (LFGP) populations (30 populations in total) to three levels of bidirectional gene flow (none, low, high) for 22 days, and monitored the proportion of each strain every 2 days via blue‐white screening (Figure 3A).

During the 22‐day experiment, after a total count of 450,603 colonies, the results showed that bidirectional gene flow had asymmetric effects on high‐fitness‐gain lac+ populations and low‐fitness‐gain lac− populations (Figure 3C,D). In the control group, all five lac+ populations (CL) and five lac− populations (CA) grew normally (Figure 3C,D), although the populations showed a slight decrease (Figure S1; Table S3). In the low gene flow group, the proportion of white colonies in the lac− populations (LA) gradually decreased, stabilizing at about 15% by Day 14. However, the proportion of blue colonies in the lac+ populations (LL) only showed a slight decrease during the first 4 days, then quickly recovered and increased, reaching about 99% by Day 10 and remaining stable thereafter (Figure 3C,D). In the high gene flow group, the proportion of white colonies in the lac− populations (HA) rapidly and continuously decreased, dropping to 0.93% by Day 10, followed by a gradual extinction, with no white colonies detected on Days 20 and 22. In stark contrast, the proportion of blue colonies in the lac+ populations (HL), although it experienced a noticeable decline to 89% during the first 2 days, quickly recovered to 97% by Day 4, and gradually approached nearly 100% afterward (Figure 3C,D). Further statistical analysis showed that the differences in the proportion of white colonies between the three parallel groups in A medium were consistently significant (*p* < 0.05) (Table 2). However, in the L medium, the differences in the proportion of blue colonies among the three parallel groups were relatively significant in the early stages, with the differences gradually narrowing in the later stages. Notably, there was no significant difference between the HL and CL groups (Table 3).

**TABLE 2:** Statistical analysis of the proportion of white colonies among the three groups in the bidirectional gene flow experiment. The means (±SD) are based on five replicate assays.

Day	CA	LA	HA	*F*	*p*
0	1 ± 0	1 ± 0	1 ± 0	/	/
2	1 ± 0	0.588 ± 0.031^a^	0.287 ± 0.108^ab^	152.983	< 0.001
4	1 ± 0	0.549 ± 0.044^a^	0.121 ± 0.071^ab^	417.084	< 0.001
6	1 ± 0	0.485 ± 0.046^a^	0.059 ± 0.016^ab^	1201.422	< 0.001
8	1 ± 0	0.367 ± 0.048^a^	0.038 ± 0.009^ab^	1512.142	< 0.001
10	1 ± 0	0.247 ± 0.025^a^	0.009 ± 0.005^ab^	6204.001	< 0.001
12	1 ± 0	0.161 ± 0.007^a^	0.005 ± 0.001^ab^	91,616.472	< 0.001
14	1 ± 0	0.143 ± 0.015^a^	0.002 ± 0.000^ab^	19,260.101	< 0.001
16	1 ± 0	0.139 ± 0.017^a^	0.001 ± 0.000^ab^	15,616.759	< 0.001
18	1 ± 0	0.135 ± 0.017^a^	0.001 ± 0.000^ab^	16,093.282	< 0.001
20	1 ± 0	0.160 ± 0.026^a^	0.000 ± 0.000^ab^	6311.349	< 0.001
22	1 ± 0	0.144 ± 0.029^a^	0.000 ± 0.000^ab^	4839.958	< 0.001

*Note:* The superscript letters (ab) represent *p* < 0.05 as compared with groups CA and LA, respectively. A slash (“/”) represents no data available.

**TABLE 3:** Statistical analysis of the proportion of blue colonies among the three groups in the bidirectional gene flow experiment. The means (±SD) are based on five replicate assays.

Day	CL	LL	HL	*F*	*p*
0	1 ± 0	1 ± 0	1 ± 0	/	/
2	1 ± 0	0.982 ± 0.007^a^	0.899 ± 0.017^ab^	133.077	< 0.001
4	1 ± 0	0.971 ± 0.019^a^	0.978 ± 0.007^a^	8.611	0.005
6	1 ± 0	0.980 ± 0.002^a^	0.984 ± 0.001^ab^	353.908	< 0.001
8	1 ± 0	0.983 ± 0.008^a^	0.992 ± 0.005^ab^	11.094	0.002
10	1 ± 0	0.990 ± 0.004^a^	0.996 ± 0.003^ab^	17.783	< 0.001
12	1 ± 0	0.987 ± 0.004^a^	0.998 ± 0.001^b^	43.277	< 0.001
14	1 ± 0	0.993 ± 0.002^a^	0.999 ± 0.001^b^	35.459	< 0.001
16	1 ± 0	0.989 ± 0.003^a^	0.999 ± 0.000^b^	51.957	< 0.001
18	1 ± 0	0.990 ± 0.003^a^	1.000 ± 0.001^b^	55.945	< 0.001
20	1 ± 0	0.987 ± 0.003^a^	1.000 ± 0.001^b^	88.849	< 0.001
22	1 ± 0	0.988 ± 0.003^a^	1.000 ± 0.000^b^	86.277	< 0.001

*Note:* The superscript letters (ab) represent *p* < 0.05 as compared with groups CL and LL, respectively. A slash (“/”) represents no data available.

Our bidirectional gene flow experiment results show that, in the absence of gene flow, both the high‐fitness‐gain lac+ populations and the low‐fitness‐gain lac− populations can grow normally. However, when gene flow is present, the low‐fitness‐gain lac− populations undergo severe population decline and/or extinction. In contrast, gene flow has no significant impact on the high‐fitness‐gain lac+ populations. Therefore, our experimental results strongly support the idea that, in the presence of bidirectional gene flow, HFGP tends to competitively exclude LFGP.

### 2.3 Demographic Effects Account for the Decline of Low‐Fitness‐Gain Populations

Our results from the above bidirectional gene flow experiment show that low‐fitness‐gain lac− populations suffered decline and even extinction. We will next identify the causes behind this. One possibility is that, in A‐medium, the introduced lac+ have higher fitness than lac−. To test this possibility, we conducted a relative fitness test. Considering the decline in some population sizes, we calculated the selection rate constant (*r*_*ij*_), which is used to measure relative fitness during population decline (Lenski et al. 1991; Travisano and Lenski 1996). The results showed that in A‐medium, when the lac+ to lac− ratio was 10:1 or 1:10, the fitness of lac− was relatively higher than that of lac+, although the difference was not significant (*p* > 0.05, Table 4). Therefore, our results indicate that the decline of lac− in A‐medium is not due to their low fitness. Since the decline of lac− populations is not due to the relatively low fitness of lac−, one major cause may be the relatively large population size of exogenous lac+ immigrants. This indicates that when the number of exogenous immigrants is relatively high (demographic effects), it creates strong competition for the latter, leading to their extinction, even if the native mutations have relatively high fitness. This is consistent with the traditional view that gene flow impedes local adaptation (E. Mayr 1963; Kirkpatrick and Barton 1997; Lenormand 2002; Bridle and Vines 2007).

**TABLE 4:** Statistical analysis of the selection rate constant. The means (±SD), based on 10 replicate assays, are presented. For L‐medium, the selection rate constant of lac+ relative to lac− was presented, and for A‐medium, the selection rate constant of lac− relative to lac+ was calculated.

Culture medium (blue:white ratio)	No. of replicates	Selection rate constant	*t*	*p*
L (10:1)	10	1.05 ± 0.60	5.506	< 0.001^a^
L (1:10)	10	1.50 ± 0.92	5.175	0.001^a^
A (10:1)	10	0.09 ± 0.31	0.950	0.367
A (1:10)	10	0.13 ± 0.33	1.200	0.261

*Note:* The superscript letter (a) represents our published data (Wu and Wu 2024).

**FIGURE 4:**
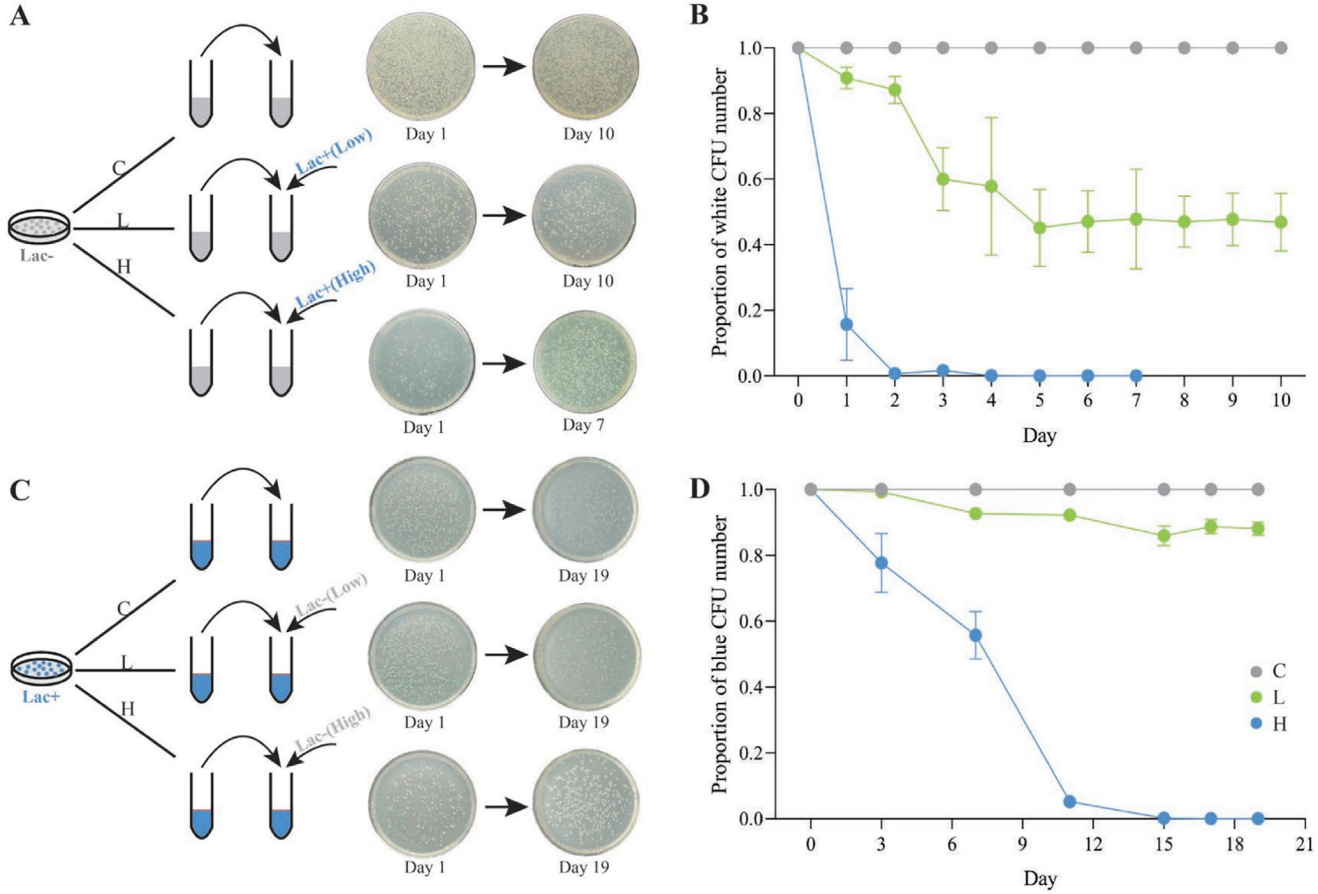
The effect of unidirectional gene flow on population survival. (A) Gene flow from lac+ to lac− with three different experimental treatments: The C group with no lac+ added, the L group with a low level of lac+ added, and the H group with a high level of lac+ added. The representative plating results from the first day and at the end of the experiment for each group are shown. (B) The change in the proportion of white colonies (lac−) over time for the three groups. (C) Gene flow from lac− to lac+ with three different experimental treatments: The C group with no lac− added, the L group with a low level of lac− added, and the H group with a high level of lac− added. The representative plating results are shown. (D) The change in the proportion of blue colonies (lac+) over time for the three groups. Each treatment had 10 biological replicates, giving a total of 30 populations for both (A) and (C). For each time point, the mean and standard deviation (calculated from the 10 replicate populations) are plotted.

To test whether demographic effects drive population decline, we performed unidirectional gene flow experiments in both directions (Figure 4). In A‐medium, lac− has a slight fitness advantage over lac+ (Table 4). When large numbers of lac+ were introduced daily into lac− populations (H group), lac− became undetectable by Day 5; additional plating on Day 7 confirmed no lac− recovery. With low introduction (L group), lac− stabilized at ~50%; without introduction (C group), lac− survived normally (Figure 4A,B; Table S4). Conversely, in L‐medium, lac+ has a large fitness advantage over lac− (Table 4). When large numbers of lac− were introduced into lac+ populations (H group), lac+ went extinct by Day 17; additional plating on day 19 confirmed no lac+ recovery. With low introduction (L group), lac+ stabilized at ~90%; without introduction (C group), lac+ survived normally (Figure 4C,D; Table S5). In both directions, the differences among the three groups were highly significant (*p* < 0.001). These results confirm that a high immigrant‐to‐resident ratio can drive native populations to extinction, regardless of their relative fitness advantage.

### 2.4 Experimental Evolutionary Evidence for Spatial‐Scale Evolutionary Bias

**FIGURE 5:**
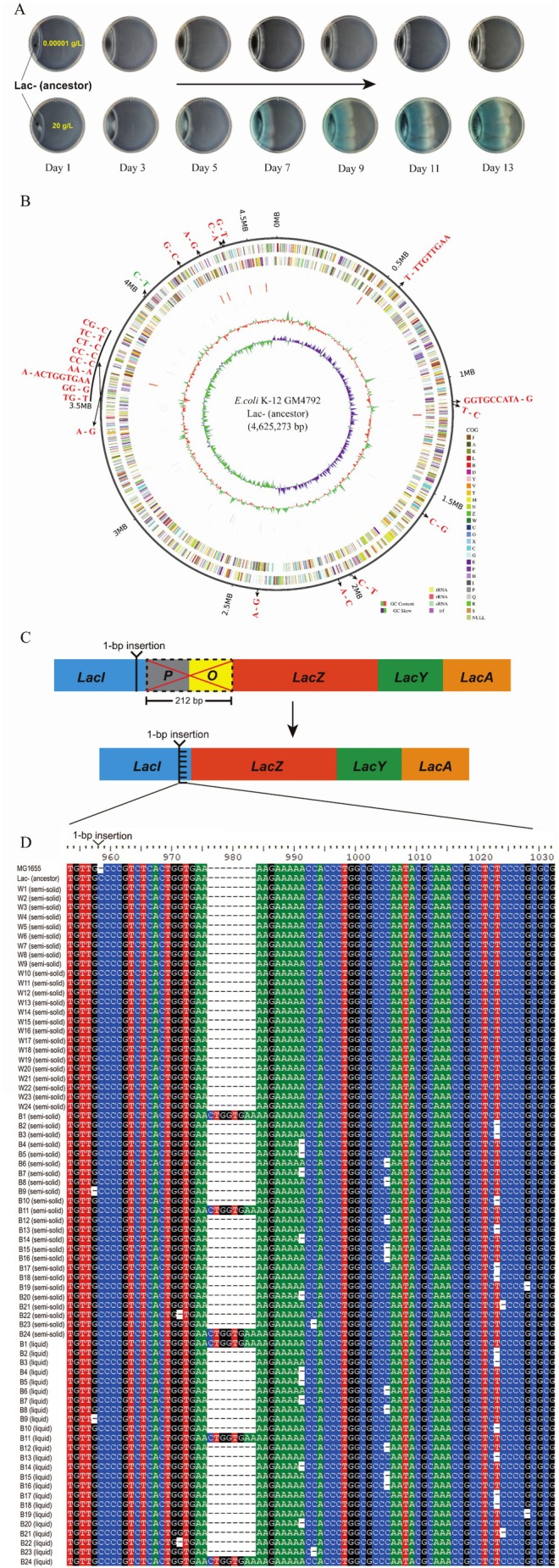
Growth and spread of the ancestral strain lac− (ancestor) on Blue plates and White plates, along with the mutation sites identified by genome sequencing. (A) The ancestral strain lac‐(ancestor) was inoculated into the liquid medium of both Blue plate (below the arrow) and White plate (above the arrow), with 24 biological replicates for each plate type. After 13 days, the ancestral strain evolved into lac+ and spread to the semi‐solid medium on the Blue plate, while no evolution into lac+ occurred on the White plate. (B) All SNPs and indels detected by genome sequencing are shown. The SNPs and indels found in 24 clones of Blue plate are shown in red, and those from White plate are shown in green. A circular map of the genome of the ancestral strain lac‐(ancestor) is displayed. From outer to inner: Genome size, forward strand genes, reverse strand genes, forward strand ncRNA, reverse strand ncRNA, repeat sequences, G + C content, and GC‐skew. The forward and reverse strand genes are colored according to the Cluster of Orthologous Groups (COG) classification. (C) The changes in the lactose operon gene structure of the strain *E. coli* K‐12 GM4792 used in our study, modified from our published study (Wu and Wu 2024). (D) Sequence alignment of gene fragments from blue monoclonal colonies on 24 Blue plates and their mutation sites. For convenience, the corresponding fragment sequences of lac‐(ancestor) and *E. coli* K‐12 MG1655 are included.

Our competitive experimental results indicated that HFGP tends to competitively exclude LFGP under gene flow. This competitive exclusion makes it more likely for species to evolve toward areas with relatively higher fitness gains, that is, SSEB. To verify the occurrence of SSEB, we conducted a comparative experimental evolution study using two types of plates (Blue and White plates; Figure 5A). Each plate consisted of a liquid medium (A‐medium with sodium acetate) and an adjacent semi‐solid medium, ensuring high gene flow. The semi‐solid medium of the Blue plate contained a high concentration of lactose (20 g/L), while that of the White plate contained a very low concentration (0.00001 g/L). The ancestral lac− strain (unable to utilize lactose) was inoculated into the liquid medium of both plates. X‐gal and IPTG were added to the semi‐solid medium to visualize lactose‐utilizing mutations (blue colonies). Each group had 24 replicates. According to SSEB, we would expect that the ancestral lac− strain is more likely to adaptively evolve to colonize the Blue plate and less likely to adaptively evolve to colonize the White plate.

As expected, after 13 days of experimental evolution, our results showed that in all 24 Blue plates with a high concentration of lactose, the ancestral bacteria underwent an evolutionary shift toward lactose utilization, and the blue colonies almost completely dominated the entire plate (Figure 5A, Figure S2). In contrast, on the 24 White plates with a low concentration of lactose, no blue colonies were observed. Instead, only a few white colonies were observed at the edge of the semi‐solid medium adjacent to the liquid medium (Figure 5A, Figure S2), which could be due to slight diffusion of sodium acetate from the liquid medium into the semi‐solid medium. This suggests that no lactose‐utilizing mutations evolved on the low‐concentration lactose plates. To further validate our observations, we performed additional sampling and plating of both the liquid and semi‐solid media from all plates (Figure S3), and counted the number of blue and white colonies. The results, based on the statistical analysis of 59,905 single‐clone colonies, showed that in the liquid and semi‐solid media of the Blue plate, blue colonies accounted for 55.9% and 91.4%, respectively, while no blue colonies were found on the White plate (Figure S4).

We identified the molecular basis of lactose utilization evolution on the Blue plates. To do this, we randomly selected one blue single‐clone colony from the semi‐solid medium of each of the 24 Blue plates, and one white single‐clone colony from the semi‐solid medium of each of the 24 White plates, and performed whole‐genome sequencing (Table S6) as well as sequencing of the lactose operon gene region. By comparing with the genome of the ancestral strain lac‐(ancestor) (Table S7), which was published previously (Wu and Wu 2024), the results showed that all 48 sequenced single‐clone colonies contained a 212 bp deletion, indicating that they all derived from the strain used in our experiment, thus ruling out the possibility of external contamination. Our results show that the genome of 24 clones from White plates is almost identical to that of the ancestral strain, except for a synonymous substitution (C‐T) (Tables S8 and S9, Figure 5B). For the 24 clones from Blue plates, almost all clones have 1–2 SNPs, except for three clones (B1, B19, and B21) that have no SNPs detected. In total, 10 types of SNPs, including both synonymous and nonsynonymous substitutions, were detected (Tables S8 and S9, Figure 5B). More importantly, we found that all 24 clones from Blue plates contain indels within the lactose operon gene, and these indels were confirmed by our PCR amplification and sequencing. Further analysis shows that these indels are located near the 1‐bp insertion site in the lactose operon region, including single‐base deletions or insertions of 8 consecutive bases (Figure 5C,D, Figure S5). These mutations restored the frameshift mutation in the ancestral strain, making it capable of utilizing lactose. Three of the 24 clones (B1, B11, and B24) had the same 8‐base insertion, while the other 21 clones had single‐base deletions at 8 different loci (957, 971, 991, 993, 1005, 1023, 1024, and 1028) (Figure 5D). In total, we identified 9 lac+ mutation types. The different mutation sites in the lac+ colonies from the various plates indicate that they underwent parallel evolution toward lactose utilization. To further determine whether the lac+ mutations on each Blue plate were of a single origin or multiple origins, we sequenced the relevant gene fragments containing these mutation sites from blue single‐clone colonies in the liquid medium of the 24 Blue plates. The results showed that the sequences were identical to those from the semi‐solid medium (Figure S5), indicating that the lac+ mutations in each of the Blue plates originated from a single source.

In the two types of plates used in our experiment, lac+ evolved on the Blue plates but did not evolve on the White plates. This may be due to differences in the relative fitness of lac+ on these two types of plates. To test this, we conducted an analysis of relative fitness. We paired the nine types of lac+ mutations detected with lac‐(ancestor) to form nine competitive pairs, and placed them in the liquid medium section of both Blue and White plates for a 24‐h competition experiment. Our results showed that in the liquid medium section of the Blue plates, regardless of whether the ratio of lac+ to lac− was 10:1 or 1:10, the relative fitness of lac+ was significantly higher than that of lac− (blue:white = 10:1, *t* = 11.45, df = 9, *p* < 0.0001; blue:white = 1:10, *t* = 8.44, df = 9, *p* < 0.0001). In contrast, in the liquid medium section of the White plates, the relative fitness of lac+ was significantly lower than that of lac− (blue:white = 10:1, *t* = −3.19, df = 9, *p* < 0.05; blue:white = 1:10, *t* = −3.01, df = 9, *p* < 0.05) (Table 5). This suggests that the lac+ mutation, which can utilize lactose, has a higher fitness gain than the ancestral strain, which uses sodium acetate, on the Blue plates. As a result, the ancestral strain evolved and spread toward the lactose‐rich area. In contrast, the lac+ mutation had relatively lower fitness gains compared to the ancestral strain on the White plates, so the ancestral strain did not evolve or spread into the lactose‐poor area. These results indicate that species are more likely to evolve and spread toward areas with higher fitness gains and less likely toward areas with lower fitness gains. Alternatively, the evolution of lac+ on the Blue plates but not on the White plates may be due to differences in mutation rates; however, since the same ancestral strain was used for both plates, this possibility is unlikely. Another possibility is that the lactose concentration in the semi‐solid medium on the White plates was too low for *E. coli* to grow. To rule out this possibility, we inoculated lac+ onto the semi‐solid medium on the White plates. The results showed that lac+ was able to grow and fully occupy the entire plate within 5 days (Figure S6). These results suggest that the lack of adaptive evolution of the ancestral strain toward lactose utilization on the White plates is more likely due to relatively lower fitness gains. Our results demonstrate that fitness gains play a crucial role in the evolutionary expansion of populations. Populations are more likely to evolve and spread toward areas with relatively higher fitness gains and less likely to evolve and spread toward areas with relatively lower fitness gains, which strongly supports the existence of SSEB.

**TABLE 5:** Statistical analysis of the selection rate constant. A one‐sample *t*‐test with a two‐tailed probability was applied, assuming the null hypothesis that the selection rate constant is zero. The selection rate constants for lac+ in comparison to lac− were computed, with the results presented as means (±SD) from nine replicate assays.

Culture medium (blue:white ratio)	No. of replicates	Selection rate constant	*t*	*p*
Blue plate (10:1)	9	1.30 ± 0.34	11.45	3.054e‐06
Blue plate (1:10)	9	0.78 ± 0.28	8.44	2.969e‐05
White plate (10:1)	9	−0.17 ± 0.16	−3.19	0.013
White plate (1:10)	9	−0.06 ± 0.06	−3.01	0.017

## 3 Discussion

The latitudinal diversity gradient is widely observed across different biological groups, but its underlying mechanisms have long been debated. In this study, we used *Escherichia coli* as a model system to demonstrate the occurrence of SSEB—a mechanism we propose may contribute to the LDG. Our experiments yielded three main findings: first, HFGP exhibits rapid population growth and spatial expansion compared to LFGP (Figure 2, Table 1); second, when gene flow is relatively high, HFGP tends to competitively exclude LFGP, while the gene flow from LFGP has very limited impact on HFGP (Figure 3; Tables 2 and 3); third, experimental populations are more likely to evolve and expand toward areas with higher fitness gains than toward areas with lower fitness gains (Figure 5). Together, these results strongly support the existence of SSEB.

Our findings are consistent with a long history of theoretical and empirical research. Early theoretical studies predicted that in spatially heterogeneous environments, natural selection favors adaptations to more productive habitats over poorer ones, leading to biased evolution toward favorable source habitats (Holt and Gaines 1992; Kawecki 1995; Kawecki et al. 1997; Holt 2003). Population genetics theory has long emphasized that asymmetric gene flow from large, central populations can hinder local adaptation in smaller, peripheral populations through genetic swamping or disruption of local adaptation (Haldane 1956; E. Mayr 1963; Kirkpatrick and Barton 1997; Lenormand 2002; Bridle and Vines 2007). This “centre–periphery” model has received empirical support from studies on walking‐sticks (Bolnick and Nosil 2007), great tits (Postma and van Noordwijk 2005), and threespine stickleback (Hendry et al. 2002). Our experiments directly mirror these predictions: HFGP, which resemble “central” populations with high density and growth, suppressed LFGP (“peripheral” populations) through asymmetric gene flow and demographic effects. Moreover, our unidirectional gene flow experiments (Figure 4) explicitly demonstrated that the magnitude of the negative impact depends on the ratio of immigrant to resident population size, a key prediction of theoretical models. Thus, our study provides controlled experimental validation of mechanisms that have long been hypothesized but rarely tested in such a direct manner.

**FIGURE 6:**
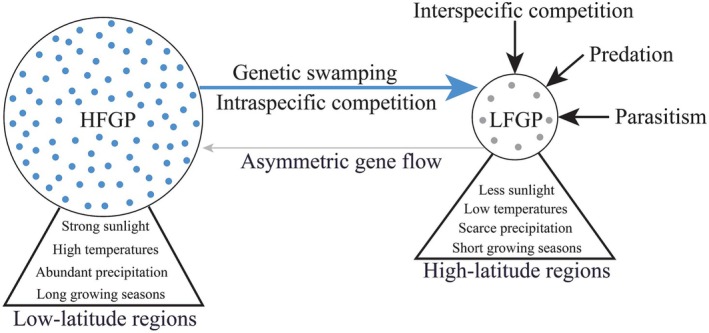
Illustration of the SSEB to account for the LDG. Climate‐related conditions make low‐latitude regions more productive than high‐latitude regions. Correspondingly, the populations in low‐latitude regions are referred to as high‐fitness‐gain geographic populations (HFGP), while those in high‐latitude regions are referred to as low‐fitness‐gain geographic populations (LFGP). The HFGP is large and abundant, while the LFGP is small and sparse. Migration from the HFGP to the LFGP exceeds migration in the reverse direction, exposing the LFGP to high levels of intraspecific competition and genetic swamping from migrants, leading to a decrease in its fitness. This reduction in fitness further makes the LFGP more vulnerable to extinction under pressures such as interspecific competition, parasitism, and predation from co‐occurring species. The asymmetrical influence between the HFGP and LFGP suppresses the adaptive evolution of the LFGP, ultimately making it less likely for low‐latitude species to adaptively evolve toward high‐latitude regions, and more likely for high‐latitude species to evolve and migrate toward low‐latitude regions. Both of these processes contribute to the formation of the LDG.

It should be noted that the strains used in our study are asexual. For sexually reproducing organisms, in addition to competition, the negative effects of hybridization, such as genetic swamping, could further reduce the adaptation of LFGP (E. Mayr 1963; Kirkpatrick and Barton 1997; Lenormand 2002; Bridle and Vines 2007). As the adaptability of LFGP declines, this could further disadvantage them in interactions with other co‐distributed species, such as competition, parasitism, and predation pressures (Sexton et al. 2009), making them more likely to be eliminated. Therefore, intraspecific competition, hybridization, and negative interspecies interactions are likely to contribute to the decline in the adaptability or extinction of LFGP, making species more likely to evolve adaptations toward areas with higher fitness gains (Figure 6). These findings suggest that, in spatially heterogeneous environments, adaptation to areas with lower fitness gains is more likely to be suppressed, leading species to preferentially evolve adaptations toward areas with higher fitness gains, thereby demonstrating the SSEB.

Given the existence of SSEB, we can extend our findings to natural ecosystems. From the poles to the equator, climatic conditions such as temperature, sunlight, precipitation, and growing season length become increasingly favorable for growth and reproduction (Hutchinson 1959; Connell and Orias 1964; Wright 1983; Turner et al. 1987; Primack 2010; Brown 2014). Consequently, for many taxa, fitness gains are expected to increase from high to low latitudes, creating a latitudinal fitness gradient. Under such a gradient, SSEB leads to two testable predictions: (1) species in low latitudes are less likely to undergo adaptive evolution toward higher latitudes; (2) species in high latitudes will tend to undergo evolutionary migration (i.e., range expansion driven by adaptive evolution rather than mere demographic dispersal) toward lower latitudes. A large body of research supports these two predictions. Below, we will provide an overview of this evidence.

### 3.1 Prediction 1: Species in Low Latitudes Are Less Likely to Undergo Adaptive Evolution Toward Higher Latitudes

This prediction can be directly derived from SSEB. According to SSEB, high‐fitness‐gain populations (e.g., those in low latitudes) tend to competitively exclude low‐fitness‐gain populations (e.g., those in high latitudes) under gene flow. Asymmetric gene flow from low‐latitude to high‐latitude populations introduces a large number of low‐latitude individuals into high‐latitude populations, overwhelming local adaptation and preventing adaptive evolution toward colder environments. Consequently, low‐latitude species rarely evolve the traits necessary to survive and reproduce at higher latitudes. Thus, SSEB offers an evolutionary mechanism to explain why low‐latitude taxa seldom expand poleward.

This perspective is consistent with but distinct from the “Tropical Niche Conservatism Hypothesis,” which attributes the rarity of tropical‐to‐temperate dispersals to a lack of cold tolerance adaptations (Wiens and Donoghue 2004, 2025; Wiens and Graham 2005). Numerous studies have supported niche conservatism across diverse taxa (Pyron and Wiens 2013; Kerkhoff et al. 2014; Pyron 2014; Qian and Ricklefs 2016; Qian et al. 2017; Chiu et al. 2020; Egan et al. 2022), showing that many tropical groups (e.g., palms, crocodiles, parrots, primates) have never successfully invaded temperate regions despite ample time and dispersal ability (Wiens and Donoghue 2004). While stabilizing selection, gene flow, and genetic constraints have been proposed as causes of tropical niche conservatism (Wiens 2004; Crisp and Cook 2012), SSEB provides an alternative mechanism: asymmetric gene flow from high‐fitness low‐latitude populations actively suppresses local adaptation in high‐latitude populations. Supporting this, a study on striped ground crickets showed that asymmetric gene flow from low‐latitude to high‐latitude populations reduces the adaptability of high‐latitude populations and hinders their expansion toward higher latitudes (Fedorka et al. 2012).

### 3.2 Prediction 2: Species in High‐Latitude Regions Tend to Undergo Evolutionary Migration Toward Low‐Latitude Regions

This prediction aligns with the “Into the Tropic Museum Hypothesis” (Rivadeneira et al. 2015; Spano et al. 2016) and the “Out of the Extratropics Hypothesis” (Raja and Kiessling 2021), both of which are used to explain the LDG. These two hypotheses are based on empirical studies of marine taxa, which show that species exhibit a much greater tendency to migrate toward the tropics than to temperate regions. Additionally, the tropics have historically acted as a center for the accumulation of marine biodiversity (Rivadeneira et al. 2015; Spano et al. 2016; Raja and Kiessling 2021). This trend is also observed in terrestrial taxa (G. Mayr 2011; Condamine et al. 2012; Pyron and Wiens 2013; Kerkhoff et al. 2014; Pyron 2014; Madern and Ostende 2015; Mannion et al. 2019; Saupe et al. 2019). For instance, fossil evidence shows that many of today's tropical‐restricted bird species once had fossil stem‐group representatives in high‐latitude regions such as Europe and North America, suggesting that their geographic distributions have undergone long‐term, equatorward contraction (Hawkins et al. 2006; G. Mayr 2011; Saupe et al. 2019). Similarly, studies of crocodilian fossils show that these species were once distributed in high‐latitude regions during the Eocene but later experienced range contraction toward lower latitudes (Mannion et al. 2019). Madern and Ostende (2015) also identified a shift in mammalian biodiversity from high to low latitudes in Miocene Eurasia (Madern and Ostende 2015). Specifically, today's nonhuman primates are largely restricted to tropical or subtropical areas, whereas in the past, they had much broader distributions, including North America, as well as northern Europe and Asia, indicating a subsequent range contraction toward low latitudes (Fleagle and Gilbert 2006). For Carnivora, which have relatively high diversification rates in temperate regions compared to the tropics, their high species richness in the tropics is believed to result from successive waves of southward colonization, followed by range contractions in high‐latitude regions, supporting the “into the tropics” model (Rolland et al. 2015). Evolutionary migration from high latitudes to low latitudes is also expected to lead to high local extirpation in high‐latitude regions, as has been observed in foraminifera over the past 40 million years (Fenton et al. 2023). These findings suggest that species' dispersal from high to low latitudes is a common phenomenon, consistent with our prediction.

Regarding the causes of evolutionary migration toward the tropics, while climate change is often considered an important factor (Fleagle and Gilbert 2006; Rolland et al. 2015; Mannion et al. 2019; Saupe et al. 2019), our findings suggest that even in the absence of climate change, higher fitness gains in low‐latitude regions would still drive evolutionary migration toward the tropics due to SSEB. That is, as populations continuously disperse toward low‐latitude regions with higher fitness gains, those low‐latitude populations would continuously outcompete populations at higher latitudes with lower fitness gains, ultimately causing the geographic range of the species to shift toward lower latitudes. Another possibility is that climatic changes, such as cooling, could make fitness gains in high‐latitude regions relatively lower than those in low‐latitude regions with more suitable habitats. The widening of this fitness‐gain gradient would intensify SSEB, thereby accelerating evolutionary migration. This is supported by previous studies showing that during global warming periods in geological history, the Earth's latitudinal temperature gradient decreased, leading to a shallower LDG gradient. However, when the climate cooled, the Earth's latitudinal temperature gradient increased, deepening the LDG gradient (Mannion et al. 2014; Fenton et al. 2023).

Although SSEB may drive evolutionary migration from high latitudes toward lower latitudes, several factors may prevent this migration from occurring. We briefly discuss three such factors below: geographical barriers, higher fitness gains at high latitudes for certain taxa, and external factors that facilitate dispersal to higher latitudes.

Geographical barriers. The existence of geographical barriers can prevent related species from evolutionary migrating toward lower latitudes. Prominent geographical obstacles greatly weaken gene flow, leading to a reduced evolutionary suppression of populations from lower latitudes on those in higher latitudes, significantly slowing the pace of evolutionary migration. For example, studies have shown that the LDG of freshwater groups is less pronounced than that of terrestrial or marine groups, which is believed to be because freshwater groups are more strongly affected by barriers (Hillebrand 2004). Additionally, studies have found that the LDG trend is more evident in the America than in Eurasia and Africa. This is believed to be because the mountain ranges in the Americas run north–south, offering less resistance to north–south species dispersal, whereas the mountain ranges in Eurasia and Africa run east–west, creating significant barriers to species' north–south movement (Hillebrand 2004).

Higher fitness gains at high latitudes. Although it is generally assumed that lower latitudes have relatively higher fitness gains, this may not apply to certain taxa. When certain taxa experience higher fitness gains at higher latitudes, they will not evolve to migrate to lower latitudes due to SSEB. For example, species like penguins and whales have their diversity peaks at high latitudes (Worm and Tittensor 2018). One possible explanation is that strong competition, predation pressures, and high species diversity in lower latitudes reduce their fitness gains in these areas, preventing these groups from migrating evolutionarily toward lower latitudes. In particular, as species diversity in low latitudes, such as in the tropics, gradually accumulates, it may lead to a decrease in the resource occupancy and fitness gains of each species in the tropics. When these gains decrease to a level no longer higher than the fitness gains of species outside the tropics, the fitness gains of species in the tropics and outside the tropics reach an equilibrium. At this point, species outside the tropics may no longer undergo evolutionary migration toward the tropics. This is consistent with the biotic resistance hypothesis, which suggests that high species diversity can significantly resist the invasion of alien species (Stachowicz et al. 1999; Kennedy et al. 2002; Beaury et al. 2020; Delavaux et al. 2023; Cheng et al. 2024). Supporting this, studies on invasive species show that the number of invasive species in tropical regions is significantly lower than in temperate regions (Sax 2001; Guo et al. 2020). Thus, SSEB can explain why some taxa did not show LDG, which remains a challenge to other hypotheses.

External factors that facilitate species dispersal to higher latitudes. Factors like global climate change, wind patterns, and animal dispersal may cause species to expand toward higher latitudes. For example, bivalves have extended their ranges to higher latitudes over time, which is considered to be due to climate change (Jablonski et al. 2006, 2013). Similar findings have been reported in terrestrial crocodylomorphs (Mannion et al. 2019). Additionally, the current global warming has caused many species' distribution areas to expand toward higher latitudes or higher altitudes (Lawlor et al. 2024). These external factors can mask the SSEB that would lead species from high latitudes to migrate toward lower latitudes. However, temporary ecological northward migration caused by external factors, without adaptive evolution, will ultimately shift southward with adaptive evolution, provided that the fitness gains in the south are higher.

## 4 Conclusion

Our experimental results indicate that high‐fitness‐gain geographic populations exhibit relatively fast population expansion and tend to competitively exclude low‐fitness‐gain geographic populations, causing species to undergo adaptive evolution biased toward regions with higher fitness gains. The existence of this SSEB suggests that the geographic range of species can evolve to track regions with high fitness gains, providing important insights into the formation of the LDG. Specifically, as long as there is a latitudinal gradient in resource distribution, SSEB is likely to suppress adaptive evolution of low‐latitude species toward higher latitudes, while also making high‐latitude species more prone to evolve and migrate toward low latitudes. Both factors would lead to an increasing concentration of species in the tropics, making tropical regions a major attractor of species diversity. Thus, SSEB may play an important role in generating the LDG, although other mechanisms may also contribute. Future work should test the generality of SSEB across taxa and examine how it interacts with classic LDG drivers.

## 5 Materials and Methods

### 5.1 Bacterial Strain

We used *E. coli* K‐12 GM4792 as the subject of this study. The strain obtained was subjected to three consecutive streaks for single‐colony isolation, and the third single‐colony culture was used as the ancestor strain, lac‐(ancestor). This strain cannot utilize lactose. In our published study (Wu and Wu 2024), we placed the lac‐(ancestor) strain in a culture medium containing lactose and sodium acetate. All five experimental populations evolved mutations enabling lactose utilization, resulting in lac+ strains. In the current study, we isolated white (lac−) and evolved blue (lac+) single colonies from each of the five populations using blue‐white screening. These lac+ and lac− strains were cultured overnight in LB liquid medium and used as subjects for this study.

### 5.2 Culture Media

This experiment primarily used two types of culture media: L‐medium and A‐medium. For L‐medium, the composition of 1 L of L‐medium consisted of basic M9 salts (Na_2_HPO_4_ 6.8 g, KH_2_PO_4_ 3.0 g, NaCl 0.5 g, NH_4_Cl 1.0 g), supplemented with 2 mL of 1.0 M MgSO_4_ solution, 0.1 mL of 1.0 M CaCl_2_ solution, 10 g sodium acetate, and 20 g lactose. A‐medium is identical to L‐medium except that lactose is omitted.

### 5.3 Population Expansion Experiment

We isolated two lac− (white) clones and two lac+ (blue) clones from each of the five experimental populations in our published study (Wu and Wu 2024), a total of 10 lac− and 10 lac+ single colonies were used in this experiment. We placed both lac+ and lac− strains on semi‐solid L‐medium to observe their population expansion. The composition of the semi‐solid medium was the same as the L‐medium, except for the addition of 3.4 g agar. For blue‐white screening, X‐gal and IPTG were added to the medium, and the solution was poured into 9 cm diameter petri dishes with a medium thickness of 1.2 cm and allowed to cool for later use.

We inoculated 100 μL of each of the 10 lac+ and 10 lac− monoclonal cultures into 9.9 mL of L‐medium and incubated them for 24 h at 37°C, 220 rpm to achieve similar physiological conditions. After incubation, we adjusted the bacterial suspension's OD values to be identical through dilution. Two lac+ and two lac− strains from the same population (L1–5) were paired to form two competition pairs. Each competition pair was placed on two Petri dishes, resulting in 20 competition plates. For each competition pair, 10 μL of the acclimated bacterial suspension from both lac+ and lac− strains was inoculated 0.5 cm apart from the center of a semi‐solid medium plate. The plates were then placed in a 37°C incubator for static cultivation. The experiment lasted for 10 days. On the 10th day, the medium was noticeably drier, and the color had slightly turned yellow. Lac+ colonies appeared blue, while lac− colonies appeared white. We took photographs of the plates on the 6th and 10th days. The images were adjusted using software Autodesk Computer Aided Design (AutoCAD, version 2025) to match the actual size of the plate (9 cm in diameter), maintaining a 1:1 scale. We then traced the area contours of the blue and white colonies and measured the area using the “AREA” command.

To analyze the proportion of lac+ and lac− in the population, we selected five plates on Day 10 for analysis. The bacterial cultures on these plates were derived from five experimental populations (L1–5). For each plate, we set up a sampling strip crossing the blue and white colony distribution areas, with four sampling points (Figure 2C). The bacterial samples were placed into 10 mL of LB liquid medium and cultured in a 220 rpm, 37°C shaking incubator. After 24 h of culture, the samples were diluted and plated onto LB solid plates containing X‐gal and IPTG. After 24 h, the number of blue and white colonies was counted.

### 5.4 Bidirectional Gene Flow Experiment

We isolated one lac− (white) clone and one lac+ (blue) clone from each of the five experimental populations, for a total of 5 lac− and 5 lac+ single colonies used in this experiment. We paired one lac+ and one lac− clone from the same population to form a competitive population pair, creating 5 competition pairs in total. First, we placed these 10 single colonies in LB liquid medium at 37°C, 220 rpm, and cultured for 24 h. Then, we adjusted the OD value of the bacterial suspension in each tube using LB medium to make them equal. Next, we took 100 μL from each lac+ population and added it to 9.9 mL of L‐medium, while taking 100 μL from each lac− population and adding it to 9.9 mL of A‐medium, incubating at 37°C, 220 rpm for 24 h (Day 1). Afterward, we transferred 2 μL of bacterial suspension from each population into 9.993 mL of fresh corresponding culture medium for subculturing each day. To investigate the impact of gene flow levels on the results, we set up three parallel groups.

For the high gene flow group, during subculture, the two populations in each competition pair exchanged 5 μL of bacterial suspension during every passage. For the low gene flow group, the two populations in each competition pair exchanged 1 μL of bacterial suspension but added 4 μL of fresh medium from the other population during every passage. For the control group, the two populations in each competition pair did not exchange bacterial suspension, only adding 5 μL of fresh medium from the other population. As a result, the culture volume for each of the 30 populations across the three parallel groups was 10 mL, with the only difference between the groups being the level of gene flow. Every 2 days, we performed blue‐white screening for each population and recorded the proportion of blue and white colonies.

### 5.5 Unidirectional Gene Flow Experiment From Lac+ Population to Lac− Population

We examined the effect of the immigration of lac+ into lac− in A‐medium. To do this, we added varying amounts of lac+ to the lac− population and observed the changes in the lac− colony ratio over time. For this, we isolated two white clones (lac−) from each of the 5 experimental populations, for a total of 10 populations. Additionally, we isolated one blue clone (lac+) from one population. From these 11 monoclonal bacterial suspensions, we took 100 μL of each and added it to 9.9 mL of A‐medium for 24 h of acclimation at 37°C, 220 rpm. Next, we transferred 100 μL from each acclimated white monoclonal bacterial suspension into 50 mL plastic tubes containing 9.9 mL of fresh A‐medium, with three parallel groups for each strain, totaling 30 populations. Meanwhile, we also took 100 μL from the acclimated blue monoclonal bacterial suspension and added it to 50 mL plastic tubes containing 9.9 mL of fresh A‐medium, creating several backups. After culturing at 37°C, 220 rpm for 24 h, we measured the OD values. Based on these OD values, we adjusted the OD of all bacterial populations to the same level. We then took 100 μL from each white monoclonal bacterial suspension and added it to 9.9 mL of fresh A‐medium in 50 mL plastic tubes.

To study the effect of different immigration levels on the population, we added varying amounts of lac+ to the lac− populations across the three parallel groups. One group (H1‐10) received the highest level of lac+, with an initial blue‐to‐white ratio of 10:1 (1 mL of lac+ added) for 10 lac− populations. Another group (L1–10) received a lower level of lac+, with an initial blue‐to‐white ratio of 1:10 (10 μL of lac+ added) and 0.99 mL of fresh A‐medium supplemented. As a control, the third group (C1–10) had no lac+ added, only 1 mL of fresh A‐medium. These 30 populations were then passaged and cultured at 37°C, 220 rpm.

Every 24 h, we took 100 μL from each of these 30 populations and added it to 50 mL plastic tubes containing 9.9 mL of fresh A‐medium, along with the corresponding amount of lac+ and/or A‐medium. We performed blue‐white screening daily to count the number of blue and white colonies. The experiment lasted for 10 days, during which the lac− strains in the H group were undetectable for three consecutive days, while the proportion of lac− in the L group stabilized.

### 5.6 Unidirectional Gene Flow Experiment From Lac− Population to Lac+ Population

We examined the impact of lac− immigration on lac+ populations. To do this, we added varying amounts of lac− to the lac+ populations and observed the changes in the lac+ colony ratio over time. For this, we isolated two blue clones (lac+) from each of the five experimental populations, for a total of 10 populations. Additionally, we isolated one white clone (lac−) from one population. We transferred 100 μL from each of these 11 monoclonal strains into 9.9 mL of L‐medium and incubated them at 37°C, 220 rpm, for 24 h. Next, we transferred 100 μL from each of the blue monoclonal populations into 50 mL plastic tubes containing 9.9 mL of fresh L‐medium, with three parallel groups for each, totaling 30 populations. Simultaneously, we also transferred 500 μL from the white monoclonal population and added it to 49.5 mL of fresh L‐medium. After incubating at 37°C, 220 rpm for 24 h, the OD values were measured. Based on the OD values, we adjusted their OD values to be the same across all samples. We then transferred 100 μL of culture medium from each of the blue monoclonal populations into 50 mL plastic tubes containing 9.9 mL of fresh L‐medium.

To study the effect of varying levels of lac− migration on the lac+ populations, we added different amounts of lac− to these three parallel groups. For one of the parallel groups (H1–10) consisting of 10 lac+ populations, the highest level of lac− was added at an initial white‐to‐blue ratio of 20:1, corresponding to a volume of 2 mL of lac−. In another parallel group (L1–10) consisting of 10 lac+ populations, a lower level of lac− was added at an initial white‐to‐blue ratio of 1:20, corresponding to a volume of 5 μL of lac−, with an additional 1.995 mL of fresh L‐medium. As a control, no lac− was added to the third parallel group (C1–10) of 10 lac+ populations; only 2 mL of fresh L‐medium was added. These 30 populations were cultured at 37°C, 220 rpm.

Every 24 h, we transferred 100 μL from each of these 30 populations into 50 mL plastic tubes containing 9.9 mL of fresh L‐medium and added the corresponding amount of lac− and/or L‐medium. We performed blue‐white screening at different time points to count the blue and white colonies. The experiment lasted for 19 days, during which the H‐populations showed no detectable lac+ for two consecutive days, and the lac+ ratio in the L‐populations eventually stabilized.

### 5.7 Fitness Assay in A‐Medium

We compared the relative fitness of lac− and lac+ in A‐medium. To do this, we isolated two lac− (white) clones and two lac+ (blue) clones from each of the five experimental populations, for a total of 10 lac− and 10 lac+ single colonies used in this experiment. First, we placed these single colonies in LB liquid medium at 37°C, 220 rpm, for 24 h. Then, we took 100 μL of culture from each population and added it to 9.9 mL of A‐medium, incubating at 37°C, 220 rpm for 24 h to achieve similar physiological conditions. After 24 h, we measured the OD values of these 20 populations and adjusted the OD values to be equal for each population. Based on the OD values, we formed two competition pairs using two blue clones and two white clones from the same experimental population (L1–5) in the ratio of blue:white = 10:1 and blue:white = 1:10, giving a total of 20 competition pairs. We mixed the paired blue and white cultures, prepared 100 μL of the mixture, and added it to a 50 mL plastic tube containing 9.9 mL of A‐medium. After mixing, we immediately diluted and plated the bacterial liquid (2 plates per population), totaling 40 plates. The plates were incubated at 37°C for 24 h, followed by blue‐white screening to count the blue and white colony numbers and calculate the pre‐competition blue‐white colony density. After 24 h of competition, we repeated the same plating and counting process to calculate the postcompetition blue‐white colony density. Based on the pre‐ and postcompetition blue‐white colony densities, we calculated the selection rate constant (r_ij_), which is given by:

$$r_{\mathrm{ij}}=\frac{\ln\left[N_{i}\left(1\right)/N_{i}\left(0\right)\right]-\ln\left[N_{j}\left(1\right)/N_{j}\left(0\right)\right]}{1\;\mathrm{day}}$$

where $N_{i}\left(0\right)$ and $N_{j}\left(0\right)$ are the initial densities of the lac+ and lac−, and $N_{i}\left(1\right)$ and $N_{j}\left(1\right)$ are their corresponding densities after 24 h (1 day) (Lenski et al. 1991; Travisano and Lenski 1996).

### 5.8 Growth Curve

We isolated one blue clone (lac+) and one white clone (lac−) from each of the five experimental populations (L1‐5), for a total of 5 lac+ and 5 lac− clones used in the experiment. First, we placed the 10 single colonies in 10 mL of LB medium and cultured them at 37°C, 220 rpm for 24 h. Then, we took 100 μL of each lac− bacterial suspension and added it to 9.9 mL of A‐medium, and 100 μL of each lac+ bacterial suspension was added to 9.9 mL of L‐medium, followed by physiological acclimatization by shaking at 37°C, 220 rpm for 24 h. After physiological acclimatization, we measured the OD values of the bacterial suspensions from the 10 colonies and adjusted their starting OD values to be equal. Next, we took 350 μL of each lac− bacterial suspension and added it to the corresponding five tubes of A‐medium, adjusting the volume to 35 mL. Similarly, we took 350 μL of each lac+ bacterial suspension and added it to the corresponding 5 tubes of L‐medium, also adjusting the volume to 35 mL. The cultures were incubated at 37°C, 220 rpm for 24 h. Every 2 h, we took 1 mL from each of the 10 tubes and transferred it to sterile 1.5 mL centrifuge tubes for storage at 4°C. Once all samples were collected, we measured the OD_600_ values of each tube in sequence using a spectrophotometer (Implen N50 Touch), performing the measurement three times for each tube.

### 5.9 Experimental Evolution for Spatial‐Scale Evolutionary Bias

To test the evolutionary bias at the spatial scale, we created two types of experimental plates, each consisting of two parts: one part is a liquid medium, and the other part is a semi‐solid medium. The liquid medium in both plates is A‐medium; the difference between them lies in the lactose concentration of the semi‐solid medium. One plate has a lactose concentration of 20 g/L (Blue plate), while the other has 0.00001 g/L (White plate). The composition of 1 L of the semi‐solid medium for the Blue plate consisted of basic M9 powder (Na_2_HPO_4_ 6.8 g, KH_2_PO_4_ 3.0 g, NaCl 0.5 g, NH_4_Cl 1.0 g), supplemented with 2 mL of 1.0 M MgSO_4_ solution, 0.1 mL of 1.0 M CaCl_2_ solution, 3.5 g agar, and 20 g lactose, with X‐gal and IPTG added. The composition of the semi‐solid medium for the White plate was identical, except for the lactose content, which was 0.00001 g.

To prepare the two types of experimental plates used in the experiment, we first tilted the Petri dishes at approximately 15° and poured about 30 mL of semi‐solid medium, which had cooled to around 50°C, into each dish. After the medium solidified and cooled, a blank area about 2 cm wide, free of medium, formed at the top of the dish. We then leveled the Petri dishes, added 2.5 mL of the above liquid medium into the blank area, and subsequently added 10 μL of the ancestral bacterial culture into the liquid medium. The dishes were incubated at 37°C and left undisturbed. Every day, 0.5 mL of fresh liquid medium was added to the culture to compensate for the reduction in medium due to consumption and evaporation. Each of the two types of plates had 24 replicates.

For the preparation of the ancestral bacterial culture, we retrieved the lac‐(ancestor) glycerol stock from the −80°C freezer, streaked it on an agar plate, picked a single colony, and transferred it into LB liquid medium, which was then incubated overnight at 37°C with shaking at 220 rpm. Afterward, 100 μL of the culture was transferred into 9.9 mL of A‐medium, and cultured at 37°C with shaking at 220 rpm for 24 h before being stored for future use.

We placed the two types of experimental plates at 37°C and incubated them undisturbed. After 13 days, we observed that lac+ colonies had nearly occupied the entire Blue plate, so we concluded the experiment. We then performed blue‐white screening on the colonies from both the liquid and semi‐solid media on different plates and counted the number of blue and white colonies.

### 5.10 Fitness Assay in Blue and White Plate

We measured the relative fitness of lac+ and lac− in Blue and White plate environments. For this, we conducted a 24‐h competition experiment by mixing lac+ with the ancestral strain lac‐(ancestor). The lac‐(ancestor) glycerol stock was retrieved from the −80°C freezer, streaked on an agar plate, and incubated at 37°C. The next day, a single colony was picked and transferred into LB liquid medium and cultured overnight at 37°C with shaking at 220 rpm. Then, 100 μL of the culture was transferred to 9.9 mL of A‐medium and incubated at 37°C with shaking at 220 rpm for 24 h for physiological conditioning. For the acquisition of lac+, we selected monoclonal colonies representing 9 lac+ mutation types detected for the experiment. These monoclonal colonies were obtained from Blue plates labeled B1, B4, B6, B9, B13, B19, B21, B22, and B23. The colonies had originally been cultured in LB liquid medium, mixed with an equal volume of 50% glycerol, and stored at −80°C. After retrieval from the −80°C freezer, these colonies were physiologically conditioned under the same experimental conditions as the ancestral strain lac‐(ancestor). Next, the OD values of the conditioned lac+ and lac‐(ancestor) cultures were adjusted to be the same. Then, the two cultures were mixed at volumes of 10:1 and 1:10, and 10 μL of the mixed culture was added to the liquid portion of both Blue and White plates. After mixing, samples were immediately taken and diluted for plating. The plates were incubated overnight at 37°C, and blue‐white screening was performed to count the blue and white colonies. After 24 h of competition, the same procedure was repeated to count the colonies postcompetition and the selection rate constant was calculated.

### 5.11 Molecular Experiment and Gene Sequencing

To identify the strain and confirm the relevant mutation sites, we performed PCR amplification and sequencing of the relevant gene fragments. The primers used in the experiment were LacIF2: CATCTGGTCGCATTGGGTCA and LaczR2: CCAGTTTGAGGGGACGACGACAGT. The target monoclonal colonies were inoculated in 10 mL of LB medium and cultured overnight at 37°C with shaking at 220 rpm, followed by colony PCR amplification. The amplification system was 25 μL, which included 12.5 μL of PrimeSTAR Max premix (Takara, Beijing), 0.75 μL of Primer 1, 0.75 μL of Primer 2, 1 μL of template (colony), and 10 μL of sterile water. The PCR conditions were as follows: pre‐denaturation at 98°C for 60 s, followed by 35 cycles of: denaturation at 98°C for 10 s, annealing at 55°C for 15 s, and extension at 72°C for 10 s, with a final extension at 72°C for 2 min. After the PCR products were checked on a 1% agarose gel, they were sent for sequencing (Comate Bioscience, Changchun).

### 5.12 Whole Genome Sequencing

Target monoclonal colonies were inoculated in LB liquid medium and cultured overnight at 37°C with shaking at 220 rpm. The culture (20 mL) was then centrifuged to enrich the bacteria. For DNA extraction, CTAB was added directly to the pellet, and the mixture was incubated at 65°C with shaking (400–1400 rpm) for 60 min, then cooled to room temperature. The sample was centrifuged at 12,000 *g* for 5 min, and the supernatant was transferred to a new 2.0 mL tube. Equal volumes of Phenol/Chloroform/Isoamyl alcohol (25:24:1) were added, vortexed, and centrifuged at 12,000 *g* for 10 min. The supernatant was then transferred to a new 1.5 mL tube, and 2/3 volume of isopropyl alcohol and 1/10 volume of 3 M sodium acetate were added. The mixture was inverted gently and stored at −20°C for at least 2 h. After centrifugation at 18,213 *g* for 15 min, the supernatant was discarded. The pellet was washed with 1 mL of 75% ethanol and centrifuged again at 18,213 *g* for 3 min. The supernatant was removed, and the pellet was air‐dried for 3–5 min before being dissolved in 20–200 μL TE buffer. DNA concentration was measured using a microplate reader, and DNA integrity was assessed by agarose gel electrophoresis.

For library preparation, a certain amount of genomic DNA was fragmented and size‐selected using magnetic beads. The selected fragments were converted into blunt‐end DNA through an end‐repair reaction, followed by A‐tailing to add a single adenosine to the 3′ end. Library adapters were then ligated to both ends of the DNA. The library was amplified by PCR and underwent quality control. The final double‐stranded library was denatured to generate a single‐stranded library, which was circularized. Single‐stranded linear DNA was digested to remove it, and the circularized library was amplified using phi29 and rolling circle amplification (RCA) to generate DNA nano balls (DNBs), each carrying approximately 300 copies of the initial single‐stranded library molecule. The DNBs were loaded onto a patterned nanoarray, and sequencing reads of PE150 base length were generated using the DNBSEQ‐T7 platform (BGI‐Shenzhen, China).

### 5.13 SNP and Indel Detection

For accurate and reliable SNP and indel detection, the raw sequencing data undergo several filtering steps: (1) removal of reads with more than 40% low‐quality (≤ 20) bases, (2) removal of reads with over 1% N bases, (3) removal of adapter contamination, and (4) removal of duplicate contamination. For SNP and indel detection, the genome of lac‐(ancestor) published previously (Wu and Wu 2024) was used as the reference genome. The clean data were then mapped using BWA (Li and Durbin 2009, 2010), followed by mutation detection using the GATK optimal variation detection pipeline (McKenna et al. 2010; DePristo et al. 2011). Picard (http://broadinstitute.github.io/picard/) was used to filter duplicate reads, and GATK performed local realignment and base quality recalibration. Key evaluation metrics, such as depth, coverage, and sample ratio, were then calculated to ensure the accuracy of the results.

### 5.14 Statistical Analysis

We used R statistical software to analyze the data. For the analysis of the size differences between the blue and white areas, we employed an independent samples *t*‐test. For the analysis of the size differences of the blue and white areas on Day 6 and Day 10, we used a paired samples *t*‐test. For the analysis of the differences in the ratio of blue and white colonies among the three groups, depending on the normality of the data, we used different statistical tests. For normally distributed data, we performed an ANOVA to analyze whether there were significant differences among the three groups. If there were significant differences, we conducted post hoc comparisons using the LSD method. For nonnormally distributed data, we applied the Kruskal–Wallis test, with pairwise comparisons conducted using the Mann–Whitney *U* test and *p*‐values corrected using the Bonferroni method. For the analysis of the relative fitness differences between lac+ and lac−, we used a one‐sample *t*‐test to evaluate the two‐tailed probability of a possible deviation of the selection rate constant from the null hypothesis, which assumes that the selection rate constant equals zero, indicating equal fitness for lac+ and lac−. For the comparison of growth curves, we used an independent samples *t*‐test. For the analysis of the differences in OD values at the beginning and end of the experiment for each population, we used a paired samples *t*‐test.

## Author Contributions

**Haoyuan Wu:** conceptualization (equal), data curation (equal), investigation (equal), methodology (equal), software (equal), validation (equal), writing – original draft (equal). **Donghui Xu:** software (equal), visualization (equal). **Yonghua Wu:** data curation (equal), formal analysis (equal), funding acquisition (equal), investigation (equal), project administration (equal), resources (equal), supervision (equal), writing – original draft (equal), writing – review and editing (equal).

## Funding

This research was supported by the National Natural Science Foundation of China (grant number 32171604).

## Ethics Statement

All experimental procedures in this study were conducted in accordance with the Declaration of Laboratory Biosafety Guidance of the Northeast Normal University (approval number NENU‐202292).

## Consent

The authors have nothing to report.

## Conflicts of Interest

The authors declare no conflicts of interest.

## Supporting information


**Figure S1:** OD_600_ values of different groups on Day 2 and Day 22 in the bidirectional gene flow experiment. Each group has five replicates. The figure shows the means and standard deviations. *p < 0.05, ***p < 0.001.
**Figure S2:** Colony growth on Blue plates and White plates on Day 13 for the experimental evolution of spatial‐scale evolutionary bias. Blue plates (B1–B24) and White plates (W1–W24) each represent 24 replicates.
**Figure S3:** Representative sampling points and plating results of Blue plate (A) and White plate (B) on Day 13.
**Figure S4:** Proportion of blue colonies. (A) The proportion of blue colonies in the liquid medium of Blue plate and White plate. (B) The proportion of blue colonies on the semi‐solid medium of Blue plate and White plate. The figure shows the mean and standard deviation. W and B represent the White plate and Blue plate, respectively.
**Figure S5:** Sequence alignment of 72 single clones from the Blue plate (B1–B24) and White plate (W1–W24). Only the sequence fragment of the lactose operon containing mutations is shown. For convenience, the corresponding fragment sequences of lac‐(ancestor) and *E. coli* K‐12 MG1655 are included.
**Figure S6:** Growth of lac+ inoculated (100 μL) onto the semi‐solid medium of the White plate. After 5 days, lac+ completely occupied the entire plate. A total of five replicates were performed.
**Table S1:** Total counts of blue and white colonies at four sampling sites across five samples in the population expansion experiment.
**Table S2:** Statistical analysis of OD_600_ values for lac− and lac+ strains. Lac+ and lac− were cultured in L‐medium and A‐medium, respectively, and their OD_600_ values were measured at different time points to construct growth curves. The means (±SD) are based on five replicate assays.
**Table S3:** Statistical analysis of OD_600_ values across different groups in the bidirectional gene flow experiment. The means (±SD) are based on five replicate assays.
**Table S4:** Statistical analysis of the proportion of white colonies among the three groups in the unidirectional gene flow experiment from lac+ to lac− populations. The means (±SD) are based on 10 replicate assays.
**Table S5:** Statistical analysis of the proportion of blue colonies among the three groups in the unidirectional gene flow experiment from lac− to lac+ populations. The means (±SD) are based on 10 replicate assays.

**Table S6:** Sequencing data statistics of 48 samples.

**Table S7:** Mapping results of 48 samples with the reference genome, along with sequencing depth and coverage statistics.

**Table S8:** SNP and indel count statistics for 48 samples.

**Table S9:** SNPs of 48 samples. The genome of lac‐ancestor is used as the reference (ref) genome. SNPs are highlighted in red or yellow. Three samples (B1, B19, and B21) have no SNPs and are not shown.

## Data Availability

The genomic sequencing data from this study are available in the CNSA (China National GeneBank Sequence Archive) under the BioProject accession number CNP0008213 (https://db.cngb.org/cnsa/). The lactose operon sequences obtained in this study were deposited in the Dryad repository (https://doi.org/10.5061/dryad.ghx3ffc2s).
