# Supplementary material for: Spatial‐Scale Evolutionary Bias Sheds Light on the Latitudinal Diversity Gradient
Source: Ecol Evol. 2026 Jul 29;16(8):e74111. doi: 10.1002/ece3.74111 (PMC13420377; doi:10.1002/ece3.74111)
Supplement: Supplementary file 1 — Figure S1: OD600 values of different groups on Day 2 and Day 22 in the bidirectional gene flow experiment. Each group has five replicates. The figure shows the means and standard deviations. *p < 0.05, ***p < 0.001. Figure S2: Colony growth on Blue plates and White plates on Day 13 for the experimental evolution of spatial‐scale evolutionary bias. Blue plates (B1–B24) and White plates (W1–W24) each represent 24 replicates. Figure S3: Representative sampling points and plating results of Blue plate (A) and White plate (B) on Day 13. Figure S4: Proportion of blue colonies. (A) The proportion of blue colonies in the liquid medium of Blue plate and White plate. (B) The proportion of blue colonies on the semi‐solid medium of Blue plate and White plate. The figure shows the mean and standard deviation. W and B represent the White plate and Blue plate, respectively. Figure S5: Sequence alignment of 72 single clones from the Blue plate (B1–B24) and White plate (W1–W24). Only the sequence fragment of the lactose operon containing mutations is shown. For convenience, the corresponding fragment sequences of lac‐(ancestor) and E. coli K‐12 MG1655 are included. Figure S6: Growth of lac+ inoculated (100 μL) onto the semi‐solid medium of the White plate. After 5 days, lac+ completely occupied the entire plate. A total of five replicates were performed. Table S1: Total counts of blue and white colonies at four sampling sites across five samples in the population expansion experiment. Table S2: Statistical analysis of OD600 values for lac− and lac+ strains. Lac+ and lac− were cultured in L‐medium and A‐medium, respectively, and their OD600 values were measured at different time points to construct growth curves. The means (±SD) are based on five replicate assays. Table S3: Statistical analysis of OD600 values across different groups in the bidirectional gene flow experiment. The means (±SD) are based on five replicate assays. Table S4: Statistical analysis of the proportion of white [file ECE3-16-e74111-s001.docx]

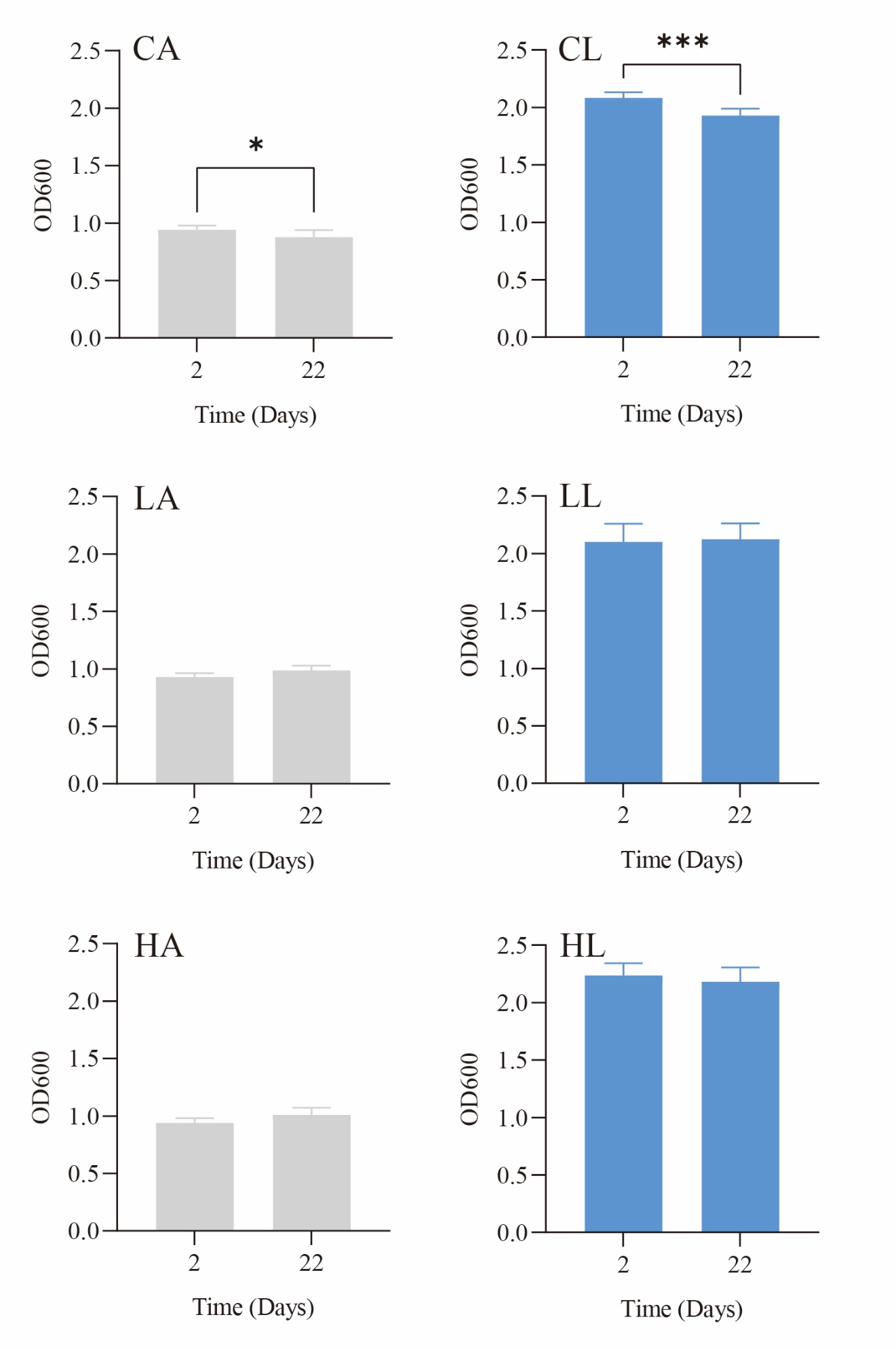


**Figure S1** OD600 values of different groups on day 2 and day 22 in the bidirectional gene flow experiment. Each group has 5 replicates. The figure shows the means and standard deviations. *p<0.05, *** p<0.001.


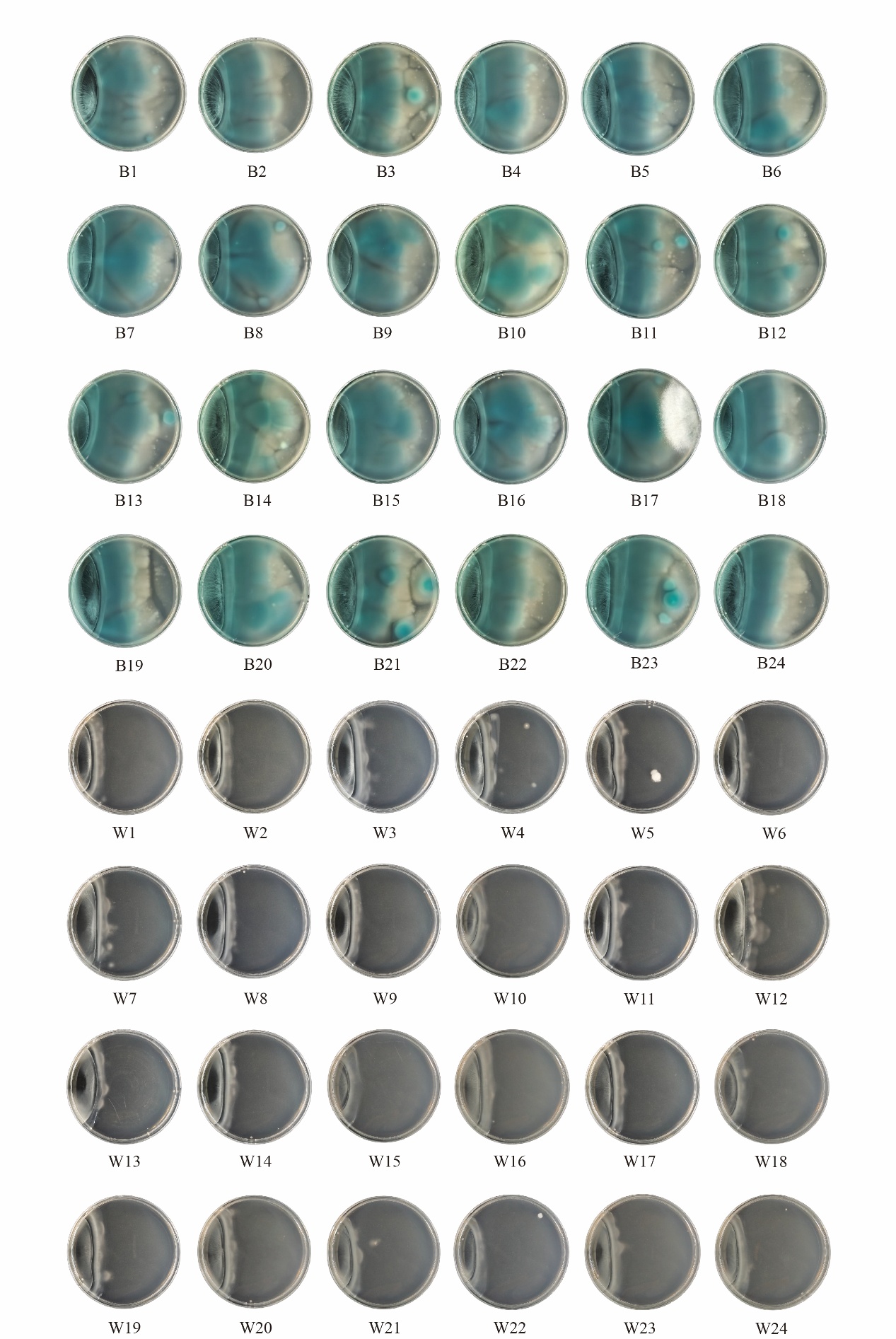


**Figure S2** Colony growth on Blue plates and White plates on day 13 for the experimental evolution of spatial-scale evolutionary bias. Blue plates (B1-B24) and White plates (W1-W24) each represent 24 replicates.


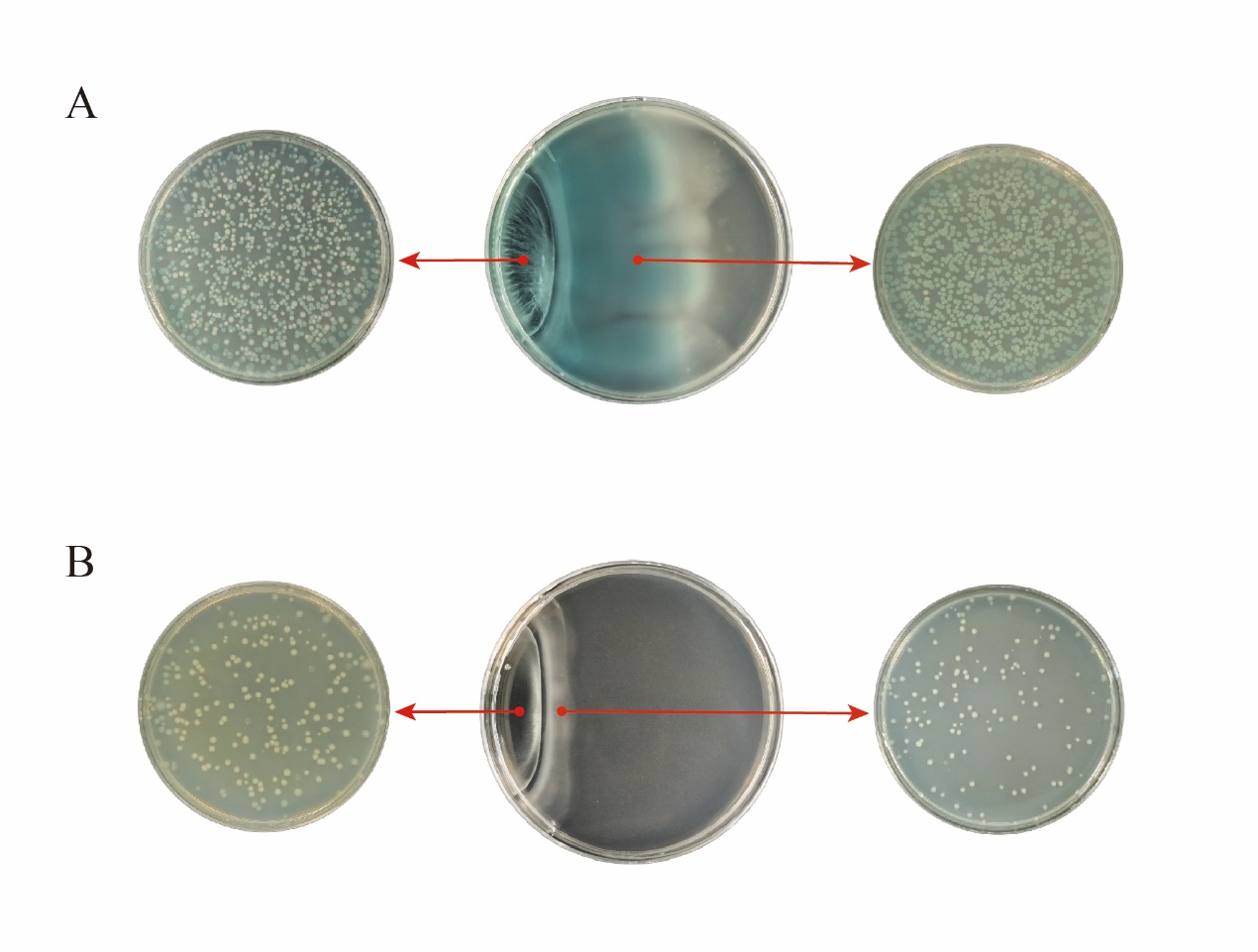


**Figure S3** Representative sampling points and plating results of Blue plate (A) and White plate (B) on day 13.


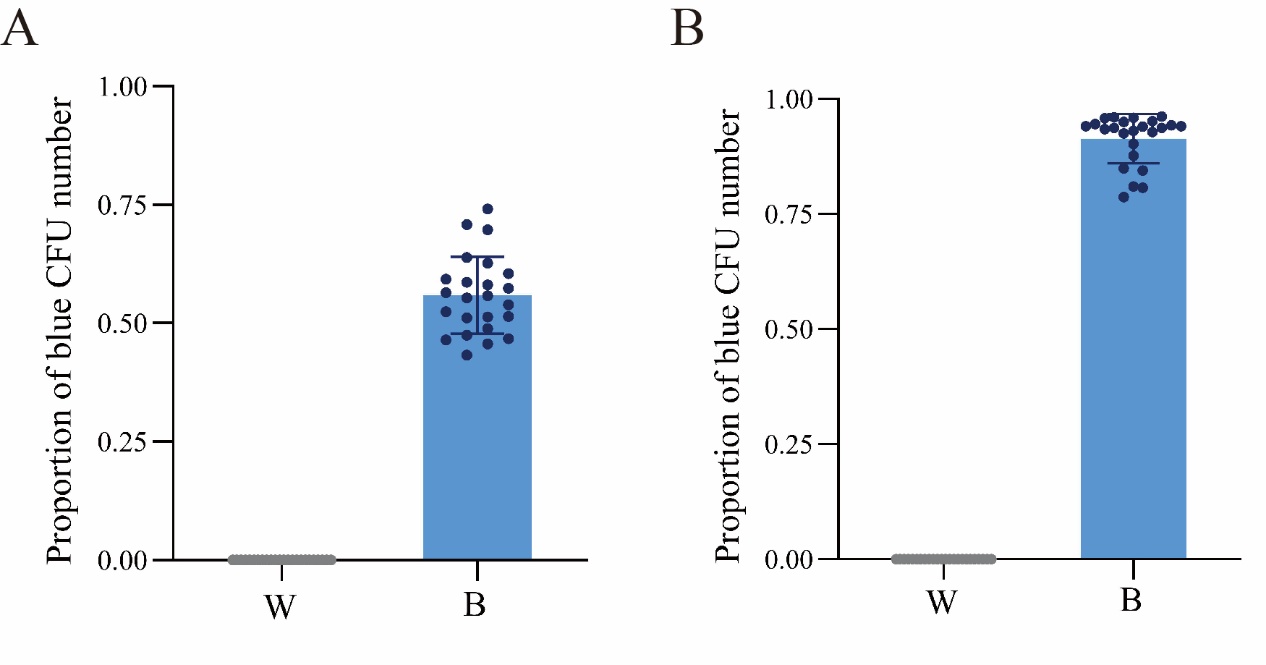


**Figure S4** Proportion of blue colonies. (A) The proportion of blue colonies in the liquid medium of Blue plate and White plate. (B) The proportion of blue colonies on the semi-solid medium of Blue plate and White plate. The figure shows the mean and standard deviation. W and B represent the White plate and Blue plate, respectively.


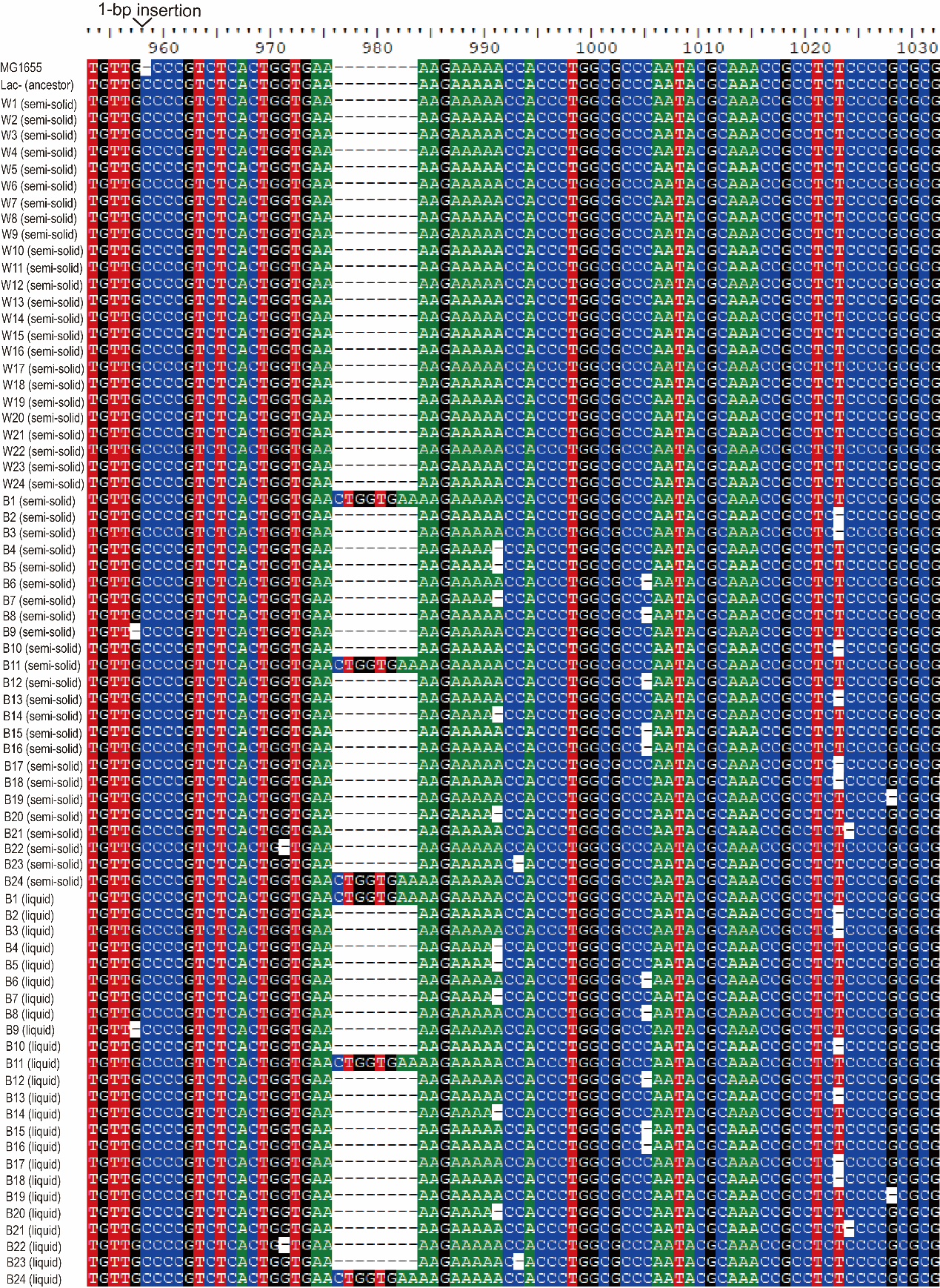


**Figure S5** Sequence alignment of 72 single clones from the Blue plate (B1-B24) and White plate (W1-W24). Only the sequence fragment of the lactose operon containing mutations is shown. For convenience, the corresponding fragment sequences of lac-(ancestor) and *E. coli* K-12 MG1655 are included.


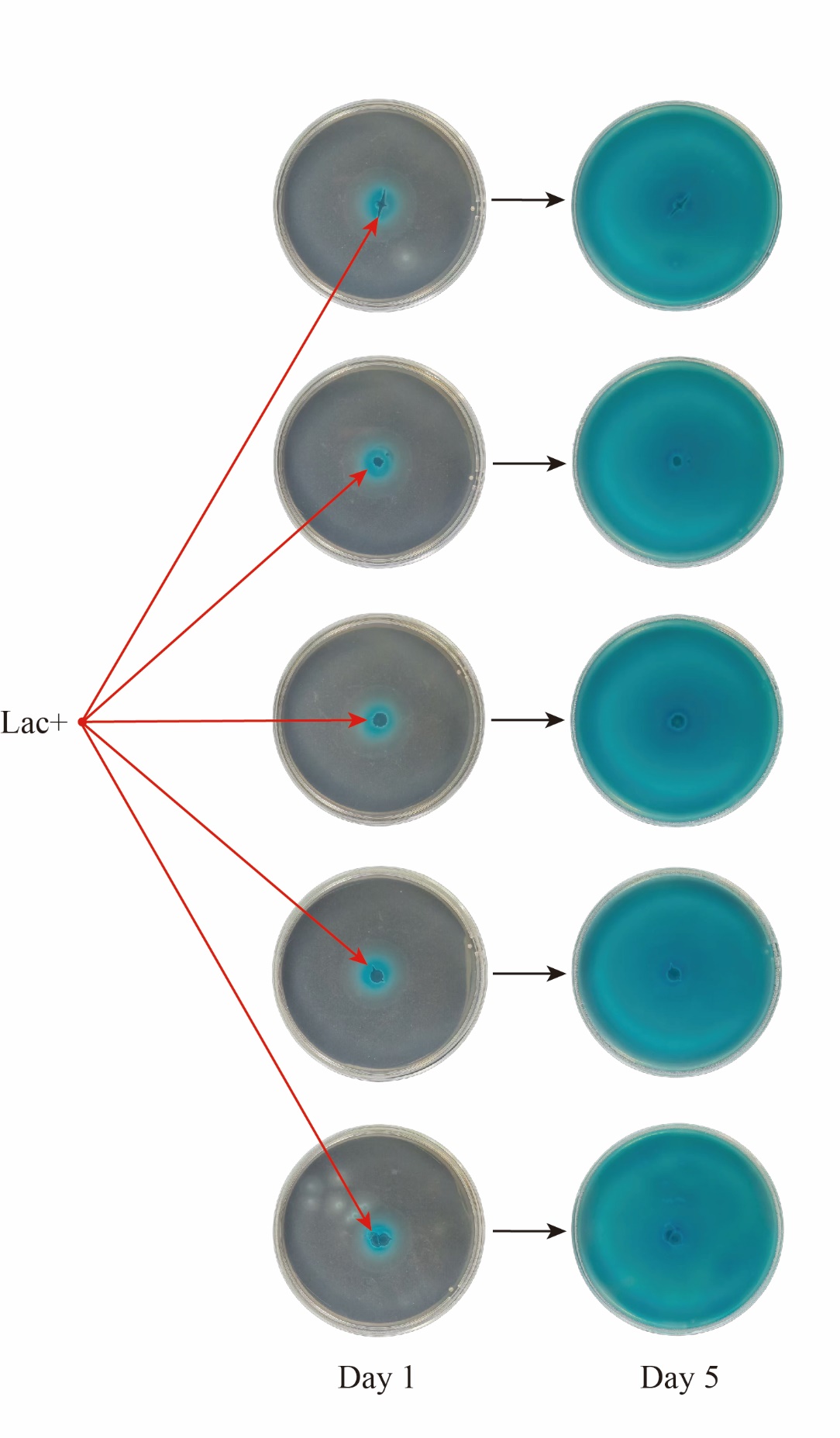


**Figure S6** Growth of lac+ inoculated (100 μl) onto the semi-solid medium of the White plate. After 5 days, lac+ completely occupied the entire plate. A total of 5 replicates were performed.

**Table S1** Total counts of blue and white colonies at four sampling sites across five samples in the population expansion experiment.

| Sampling site | No. of replicates | Blue | White |
| --- | --- | --- | --- |
| a | 5 | 2031 | 0 |
| b | 5 | 3465 | 75 |
| c | 5 | 3035 | 610 |
| d | 5 | 0 | 3452 |

**Table S2** Statistical analysis of OD600 values for lac- and lac+ strains. Lac+ and lac- were cultured in L-medium and A-medium, respectively, and their OD600 values were measured at different time points to construct growth curves. The means (± SD) are based on 5 replicate assays.

| Time | Lac- | Lac+ | t | *p* |
| --- | --- | --- | --- | --- |
| 0 | 0.011±0.002 | 0.014±0.004 | -1.835 | 0.104 |
| 2 | 0.024±0.003 | 0.024±0.006 | 0.000 | 1.000 |
| 4 | 0.041±0.005 | 0.049±0.008 | -1.768 | 0.115 |
| 6 | 0.060±0.005 | 0.117±0.011 | -11.244 | <0.001 |
| 8 | 0.117±0.014 | 0.216±0.005 | -15.109 | <0.001 |
| 10 | 0.209±0.017 | 0.434±0.052 | -9.097 | <0.001 |
| 12 | 0.306±0.014 | 0.643±0.032 | -21.473 | <0.001 |
| 14 | 0.376±0.009 | 0.929±0.014 | -74.866 | <0.001 |
| 16 | 0.443±0.009 | 1.070±0.029 | -45.242 | <0.001 |
| 18 | 0.508±0.009 | 1.239±0.018 | -81.161 | <0.001 |
| 20 | 0.583±0.009 | 1.386±0.055 | -32.257 | <0.001 |
| 22 | 0.664±0.009 | 1.513±0.103 | -18.413 | <0.001 |
| 24 | 0.745±0.011 | 1.627±0.101 | -19.411 | <0.001 |

**Table S3** Statistical analysis of OD600 values across different groups in the bidirectional gene flow experiment. The means (±SD) are based on 5 replicate assays.

| Group | Day 2 | Day 22 | df | t | *p* |
| --- | --- | --- | --- | --- | --- |
| CA | 0.942 ± 0.040 | 0.877 ± 0.040 | 4 | 3.591 | 0.022 |
| CL | 2.084 ± 0.036 | 1.929 ± 0.036 | 4 | 9.387 | 0.000 |
| LA | 0.930 ± 0.052 | 0.985 ± 0.052 | 4 | 2.382 | 0.075 |
| LL | 2.102 ± 0.106 | 2.125 ± 0.106 | 4 | 0.482 | 0.654 |
| HA | 0.940 ± 0.060 | 1.008 ± 0.060 | 4 | 2.557 | 0.062 |
| HL | 2.236 ± 0.111 | 2.180 ± 0.111 | 4 | 1.108 | 0.330 |

**Table S4** Statistical analysis of the proportion of white colonies among the three groups in the unidirectional gene flow experiment from lac+ to lac- populations. The means (± SD) are based on 10 replicate assays.

| Day | C | L | H | Z | *p* |
| --- | --- | --- | --- | --- | --- |
| 1 | 1±0 | 0.908±0.03^a^ | 0.157±0.11^ab^ | 26.796 | <0.001 |
| 2 | 1±0 | 0.872±0.04^a^ | 0.007±0.01^ab^ | 26.790 | <0.001 |
| 3 | 1±0 | 0.600±0.10^a^ | 0.016±0.01^ab^ | 26.790 | <0.001 |
| 4 | 1±0 | 0.578±0.21^a^ | 0.001±0.00^ab^ | 27.008 | <0.001 |
| 5 | 1±0 | 0.451±0.12^a^ | 0.000±0.00^ab^ | 27.851 | <0.001 |
| 6 | 1±0 | 0.471±0.09^a^ | 0.000±0.00^ab^ | 27.851 | <0.001 |
| 7 | 1±0 | 0.478±0.15^a^ | 0.000±0.00^ab^ | 27.851 | <0.001 |
| 8 | 1±0 | 0.470±0.08 | / | 16.309 | <0.001 |
| 9 | 1±0 | 0.477±0.08 | / | 16.309 | <0.001 |
| 10 | 1±0 | 0.468±0.09 | / | 16.309 | <0.001 |

*Note*: ^a, b^ represent p < 0.05 as compared with groups C and L, respectively. A slash ("/") represents no data available.

**Table S5** Statistical analysis of the proportion of blue colonies among the three groups in the unidirectional gene flow experiment from lac- to lac+ populations. The means (±SD) are based on 10 replicate assays.

| Day | C | L | H | Z | *p* |
| --- | --- | --- | --- | --- | --- |
| 3 | 1±0 | 0.993±0.01^a^ | 0.778±0.09^ab^ | 26.790 | <0.001 |
| 7 | 1±0 | 0.927±0.02^a^ | 0.558±0.07^ab^ | 26.790 | <0.001 |
| 11 | 1±0 | 0.922±0.01^a^ | 0.052±0.02^ab^ | 26.790 | <0.001 |
| 15 | 1±0 | 0.860±0.03^a^ | 0.001±0.00^ab^ | 27.320 | <0.001 |
| 17 | 1±0 | 0.888±0.02^a^ | 0.000±0.00^ab^ | 27.858 | <0.001 |
| 19 | 1±0 | 0.881±0.02^a^ | 0.000±0.00^ab^ | 27.851 | <0.001 |

*Note*: ^a, b^ represent p < 0.05 as compared with groups C and L, respectively.
